# *Cdc42* defect reveals insights into microvilli organization and function in T cell immunity

**DOI:** 10.1073/pnas.2505291122

**Published:** 2025-07-25

**Authors:** Won-Chang Soh, Sang-Moo Park, Jeong-Su Park, Hatice Karabulut, Hee-Tae Kang, Sun-Kyoung Kang, Min-Sang Kim, Jihwan Park, Sunjae Lee, Hye-Ran Kim, Chang-Duk Jun

**Affiliations:** ^a^Life Sciences and Medical Convergence Gwangju Institute of Science and Technology, Gwangju 61005, Republic of Korea; ^b^Immune Synapse and Cell Therapy Research Center, Gwangju Institute of Science and Technology, Gwangju 61005, Republic of Korea; ^c^Division of Rare and Refractory Cancer, Tumor Immunology, Research Institute, National Cancer Center, Goyang 10408, Republic of Korea

**Keywords:** Cdc42, T cell microvilli, T cell activation

## Abstract

Microvilli on T cells differ from those on epithelial cells, exhibiting filopodia-like characteristics that facilitate the clustering of molecules essential for sensing and cell migration. Recently, they have also been recognized as the structures from which T cell immunological synaptosomes (TIS) are released. In this study, we examined a key determinant of microvilli organization during T cell development and explored the functional roles of these structures, particularly in relation to T cell behaviors. During thymocyte maturation, single-positive thymocytes were found to develop more and longer microvilli than double-positive thymocytes. However, the deletion or inhibition of Cdc42, a small Rho family protein, significantly reduced both the number and length of microvilli in single-positive thymocytes, leading to decreased cell mass. This reduction in microvilli correlates with a decrease in antigen recognition, leading to diminished T cell activation and adhesion, as well as reduced TIS production, while intrinsic migratory properties remain unaffected. These findings highlight the filopodia-like characteristics of T cell microvilli. In this context, Cdc42 contributes significantly to microvilli formation, thereby shaping T cell function.

## Significance

The regulation and function of T cell microvilli remain incompletely understood. In this study, we show that Cdc42 is involved in controlling microvilli formation during the transition from the DP to SP stage, thereby enhancing antigen recognition, promoting TCR-mediated activation, and facilitating APC binding. Notably, instead of disappearing upon activation, microvilli function as scaffolds for TCR microcluster formation and serve as a source of microvesicles during immune synapse formation. By utilizing Cdc42 knockout and inhibition models, we demonstrate the crucial role of Cdc42 in orchestrating microvilli dynamics, T cell activation, and immune communication. These findings establish a previously unrecognized connection between Cdc42-mediated microvilli regulation and adaptive immunity.

Microvilli are finger-like membrane protrusions composed of cross-linked actin bundles that are present on various cell surfaces (1). The most well-characterized microvilli are those of intestinal epithelial cells, which maintain a constant length and are specialized for nutrient absorption (2, 3). T cells also have numerous microvilli on their surface (2, 3); however, unlike those on epithelial cells, T cell microvilli are highly dynamic and resemble filopodia, intermittently growing and shrinking through the assembly and disassembly of actin filaments (4, 5). Given their mobility, T cell microvilli have long been suggested to facilitate adhesion to vascular endothelial cells and, more recently, to serve as antigen sensors that interact with antigen-presenting cells (APCs) or target cells (4, 6).

Despite these established roles, how T cells initially recognize antigens on APCs remains a subject of debate. Advances in microscopy and the identification of microvillar-specific proteins have provided insights into the dynamic behavior of T cell microvilli during interactions with APCs (6, 7). Jung et al. demonstrated that TCRs are highly clustered at the tips of microvilli, positioning these structures as effective antigen sensors (8). They also reported that the ERM-dependent assembly of key TCR signaling molecules—CD4, CD2, Lck, and LAT—as well as costimulatory molecules occurs at the microvillar tips (9). Our group visualized the dynamic movement of microvilli toward the surface of opposing cells during antigen recognition, along with the specific localization of the TCR ζ-chain at the tips of microvilli (7).

However, an alternative perspective suggests that antigen recognition and early immune synapse formation are primarily driven by TCR microclusters, which are thought to form independently of microvilli (10–12). This divergence in focus raises an important question: how are microvilli and TCR microclusters functionally related? Previous studies indicate that microvilli collapse in a Rac1-dependent manner upon T cell activation (13), suggesting they may not directly contribute to TCR microcluster formation. Additionally, prevailing models of microcluster formation emphasize the random diffusion of membrane proteins, often overlooking the structural role of microvilli (14). These complementary viewpoints highlight the need for further investigation into whether microvilli actively participate in the spatial organization of TCR microclusters or merely serve as initial antigen-sensing platforms before giving way to microcluster-driven signaling.

Beyond antigen recognition, recent studies have shown that T cells release synaptic ectosomes or TCR-enriched microvesicles at the immune synapse, implicating ESCRT proteins and ectocytosis mechanisms (15, 16). However, the potential involvement of microvilli in this process has also remained largely unaddressed. Our research proposes that microvilli serve as the source of synaptic ectosomes, which detach from the T cell body upon activation and transfer to APCs (7). We have previously termed these vesicles “T cell immunological synaptosomes (TIS)” due to their ability to transfer T cell messages. Based on this, we hypothesize that microvilli play broader roles beyond antigen sensing, contributing to both the initiation and regulation of immune responses and the intercellular transmission of T cell signals.

Another question in this study is how microvilli form and undergo structural remodeling during T cell development, which remains unclear. In particular, their regulation at each developmental stage in the thymus is poorly understood, and little is known about the factors governing their formation or the consequences of their disruption. In this study, we provide the evidence that microvilli presentation is dynamically regulated throughout T cell development. Specifically, we found that double-positive (DP) thymocytes exhibit short and sparse microvilli, whereas single-positive (SP) thymocytes develop significantly longer and more numerous microvilli. Cdc42, a member of the Rho family GTPases, is a key regulator of actin dynamics, functioning alongside Rac1 and RhoA (17). It plays a particularly critical role in filopodia formation through interactions with ERM and other actin-binding proteins (18). Given the strong structural and functional similarities between T cell microvilli and filopodia, we investigated the role of Cdc42 in microvilli formation and its impact on T cell development and function.

Our findings demonstrate that Cdc42 is indispensable for proper microvilli organization during T cell maturation. Notably, we found that T cell microvilli are not merely passive antigen sensors; they actively contribute to TCR microcluster formation and facilitate their translocation to the central supramolecular activation cluster (c-SMAC), thereby reinforcing the immunological synapse. Furthermore, disruption of Cdc42—through knockout or inhibition—resulted in defective microvilli formation and impaired production of TIS in activated T cells. Despite these defects, other Rho family GTPases and actin-regulatory proteins remained functional, preserving T cell motility. These findings highlight the specific role of Cdc42 in immunological synapse formation and the specialized generation of synaptosomes, without broadly compromising other T cell functions.

## Results

### Microvilli Are Key Structures in T Cell Activation, TCR Microcluster Organization, and the Generation of Immunological Synaptosomes.

**Fig. 1.**
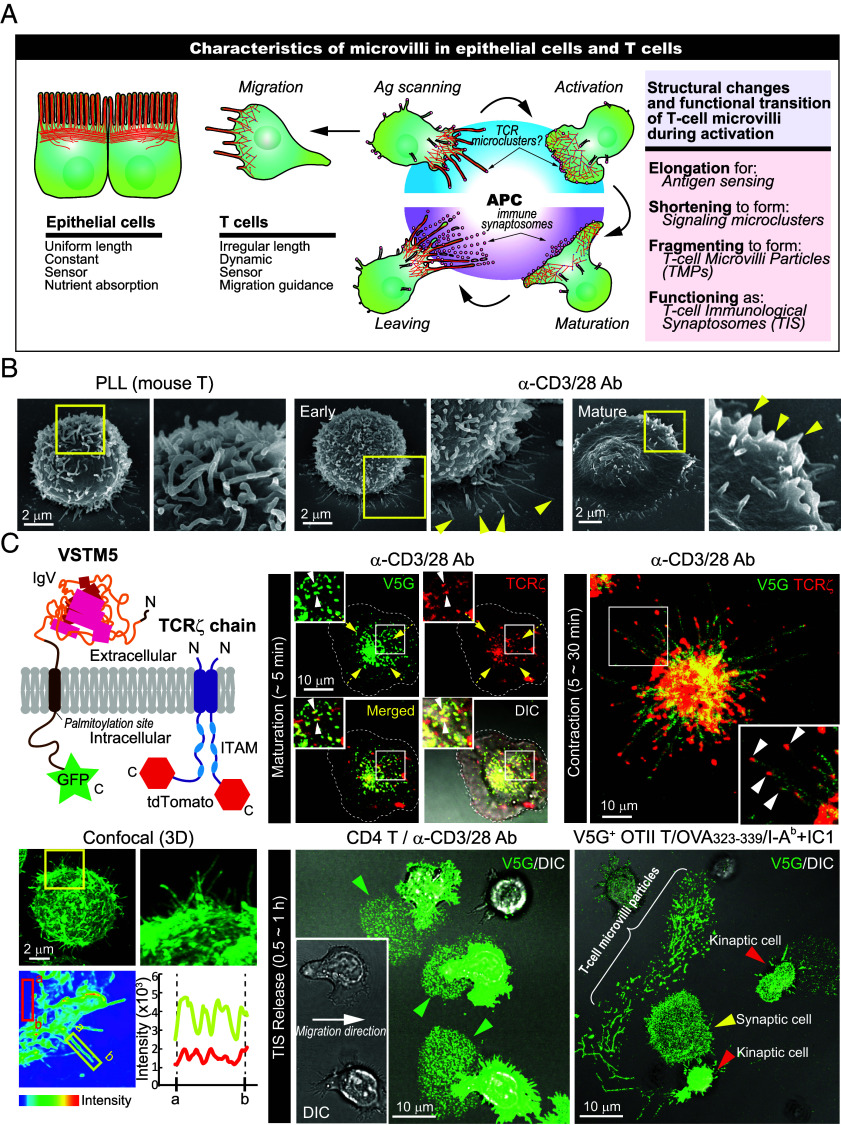
T cell microvilli are essential structures that not only scan antigens but also play a crucial role in delivering T cell messages. (*A*) Schematic diagram comparing the characteristics of microvilli between epithelial cells and T cells. (*B*) SEM images of CD4^+^ blast cells from wild-type (WT) mice in activating manner on anti-CD3/CD28-coated surfaces. Arrows indicate protruding T cell microvilli in activated T cells. (*C*) The movement of Vstm5_GFP (V5G) and TCR ζ _tdTomato signals during T cell activation on immobilized antibodies (iAb). Schematic representation of the V5G and TCR ζ _tdTomato constructs (*Top Left*) and the 3-dimensional confocal imaging of V5G (*Bottom Left*). Intensity mapping of V5G shows distinct peaks in fluorescence intensity (*Bottom Left*). The localization of V5G and TCR ζ during the maturation and contraction phases of T cells on plate-immobilized anti-CD3/CD28 (iAb) surfaces is shown. Yellow arrows indicate the direction of V5G (green) and TCR ζ (red) movement. White arrows highlight the TCR ζ located at the tips of microvilli (*Top Right*). Green arrows indicate TIS release behind moving CD4^+^ T cells expressing V5G on iAb and supported lipid bilayers presenting OVA_323-339_/I-Ab+IC1 (*Bottom Right*).

T cells are covered with numerous membrane protrusions called microvilli, which differ markedly from those on epithelial cells (Fig. 1*A*). Whereas epithelial microvilli are uniform and mainly involved in nutrient absorption, T cell microvilli show irregular, dynamic morphologies that vary with cellular state and environment (Fig. 1 *A* andd *B*). Despite their abundance, the mechanisms underlying microvilli formation and regulation in T cells remain poorly understood. Moreover, their fate during T cell activation and deactivation has not been fully explored, and it is unknown whether microvilli dynamics are regulated during thymic development.

While T cell microvilli are primarily involved in antigen sensing, we have previously demonstrated that, due to the enrichment of TCRs at their tips, microvilli serve as carriers that facilitate the movement of TCR microclusters toward the c-SMAC, thereby enhancing activation signaling and facilitating the formation of a stable immunological synapse (4, 7). This was supported by the colocalization of the microvilli-specific protein Vstm5 with TCRζ at microclusters (Fig. 1*C*) (7). Additionally, EM images of mature immunological synapses show partially intact microvilli at the synapse periphery (Fig. 1 *B*, *Right*, yellow arrows), suggesting roles in synapse formation, maintenance, and dissociation.

Previous studies from other groups have suggested that activated T cells release TCR-enriched microvesicles, or synaptic ectosomes, primarily from the c-SMAC (15, 16). However, as shown in Fig. 1*C*, during later stages of activation—particularly the contraction and final kinapse stages—TCRs remain at microvilli tips, which eventually detach to form TCR-containing microvesicles. These observations suggest that microvilli play roles beyond antigen sensing, contributing to intercellular communication through vesiculation. Fig. 1*A* summarizes the structural and functional transitions of microvilli during T cell activation, including elongation for sensing, shortening for microcluster formation, fragmentation into T cell microvilli particles, and their final role as TIS. To explore these diverse functions, we first investigated microvilli organization during T cell development. Using a Cdc42 conditional knockout model, we examined how microvilli formation is regulated, their contribution to TCR microcluster and synapse formation, and their role as a vesicle source for intercellular signaling. In some experiments, the Cdc42 inhibitor CASIN was used to control for off-target effects of genetic deletion.

### Microvillar Presentation on the Surface of Thymocytes Is Controlled During Development.

**Fig. 2.**
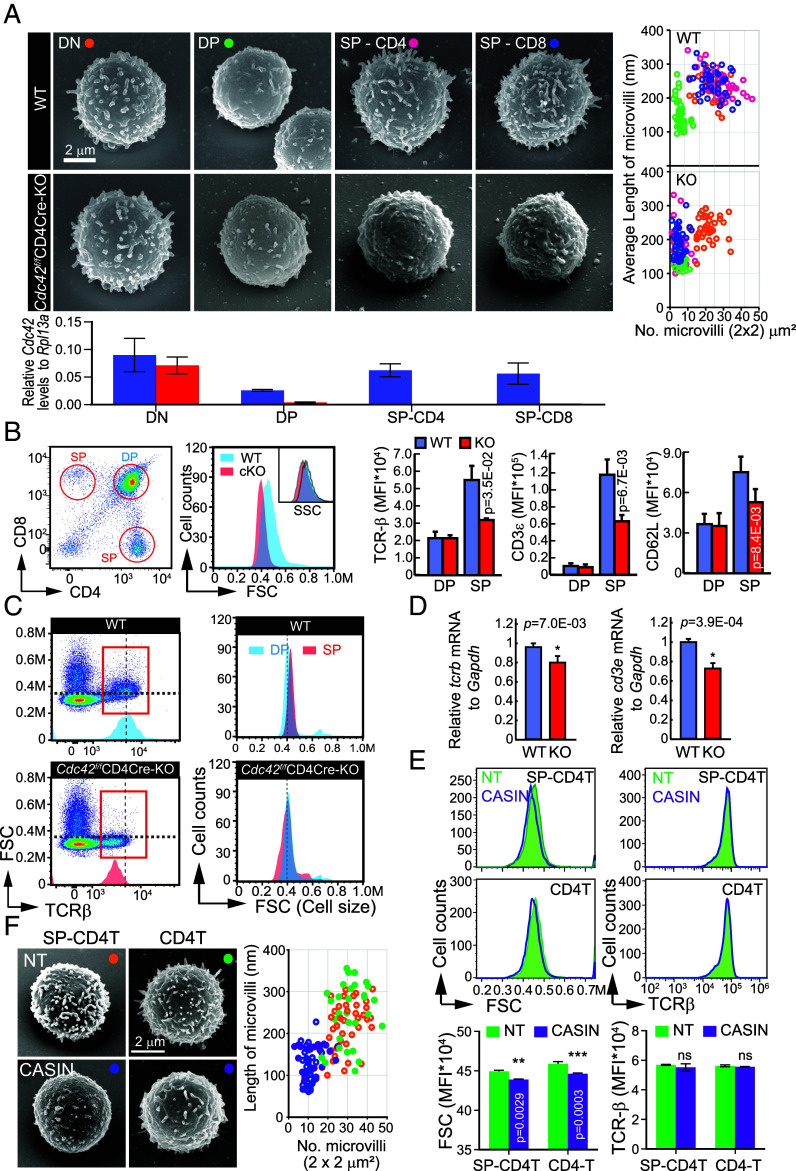
Cdc42 is required for microvilli organization on the cell surface in both thymocytes and mature T cells. (*A*) SEM images of double-negative (DN), double-positive (DP), and single-positive (SP) thymocytes from *Cdc42^f/f^* (WT) and *Cdc42^f/f^*CD4Cre (KO) mice. The number and length of microvilli per 4 μm^2^ were quantified using the ImageJ software (n = 100). Each orange (DN), green (DP), purple (CD4^+^SP), and blue (CD8^+^ SP) circle in the graphs represents an individual cell. (*B*) Flow cytometric analysis for FSC, SSC of total thymocytes and TCRβ, CD3ε, CD62L of DP, SP thymocytes gated from total thymocytes from *Cdc42^f/f^* (WT) and *Cdc42^f/f^*CD4Cre (KO) mice. (*C*) Flow cytometric analysis for FSC and SSC on TCRβ^high^ thymocytes gated from total thymocytes from *Cdc42^f/f^* (WT) and *Cdc42^f/f^*CD4Cre (KO) mice. (*D*) Expression of TCRβ, CD3ε on CD4^+^ SP thymocytes from WT and KO mice. (*E* and *F*) Surface expression of TCRβ and cell size (FSC) in CD4^+^ SP thymocytes and CD4^+^ T cells (*E*), SEM images of CD4^+^ SP thymocytes and CD4^+^ T cells (*F*) in the presence or absence of CASIN (10 µM, 2 h). (*E* and *F*) Data represent the mean ± SEM of three independent experiments. Statistics was performed using Student’s *t* test.

Although thymocytes progress through multiple developmental stages, the regulation of surface microvilli remains unclear. To address this, we analyzed sorted thymocyte subsets by SEM. In WT mice, microvilli were abundant at the DN stage, reduced at the DP stage, and restored at the SP stage. SP thymocytes also showed increased FSC and SSC, likely reflecting microvilli reappearance (Fig. 2 *A* and *B*). Intriguingly, qPCR revealed that Cdc42 expression was relatively low in DP thymocytes compared to DN and SP stages.

*Cdc42^f/f^*CD4Cre (*Cdc42*-conditional-knockout, cKO) mice were generated by crossing *Cdc42^f/f^* mice with CD4Cre transgenic mice (*SI Appendix*, Fig. S1*A*). These mice displayed normal body and immune organ sizes (*SI Appendix*, Fig. S1 *B* and *C*), but showed a reduced thymic medulla area, where CD4^+^ and CD8^+^ SP thymocytes reside (*SI Appendix*, Fig. S1*D*). While DN and DP thymocyte populations were unaffected, CD4^+^ and CD8^+^ SP thymocytes were significantly reduced (*SI Appendix*, Fig. S1*E*). Consistent with previous reports (19, 20), Cdc42 deficiency impaired T cell maturation, leading to decreased peripheral CD4^+^ and CD8^+^ T cells and increased TCRβ^−^ DN T cells (*SI Appendix*, Fig. S1*F*). Peripheral lymphoid organs also showed an expansion of γδ T cells (*SI Appendix*, Fig. S2*A*), which survived better than αβ T cells under stimulation in Cdc42-cKO mice (*SI Appendix*, Fig. S2*B*). This shift correlated with enhanced tumor suppression in a B16 melanoma model, likely due to Th17-like γδ T cells (*SI Appendix*, Fig. S2*C*) (21).

In contrast to WT cells, *Cdc42*-cKO SP thymocytes exhibited significantly fewer and shorter microvilli (Fig. 2*A*), failed to increase in size during DP-to-SP transition, and had reduced surface TCRβ and corresponding mRNA levels (Fig. 2 *B*–*D*). We also utilized *Cdc42^f/f^* dLck-Cre mice to induce Cre expression at late thymocyte stages. Although thymocyte development appeared largely normal (*SI Appendix*, Fig. S3 *A* and *B*), these mice showed defects in peripheral CD8^+^ T cells (*SI Appendix*, Fig. S3*C*) and mostly abnormal microvilli structures (*SI Appendix*, Fig. S3*D*). However, western blotting revealed inconsistent and often minimal reduction of Cdc42 protein compared to WT (*SI Appendix*, Fig. S3*C*), limiting the model’s utility. Therefore, we focused on SP thymocytes from *Cdc42^f/f^* CD4-Cre mice, which showed more complete Cdc42 deletion and clearer microvilli defects (*SI Appendix*, Fig. S1*A* and Fig. 2 *A*–*C*). Since our goal was to examine microvilli function using Cdc42 deletion, dLck-Cre peripheral T cells were used only for supplementary analysis.

Because *Cdc42* knockout results in both microvilli loss and reduced TCR expression, we sought to determine whether the diminished TCR expression in *Cdc42*-cKO cells was due to the absence of microvilli or an intrinsic defect in TCR expression during differentiation. To address this, we gated cells of similar size and evaluated their TCR expression. The persistently low TCR levels observed (*SI Appendix*, Fig. S3*D*) suggest that the *Cdc42*-cKO induces a differentiation defect, leading to reduced TCR expression independent of cell size. This diminished TCR expression complicates the interpretation of subsequent experiments, making it challenging to discern whether observed effects arise from microvilli abnormalities or defects in TCR expression. To overcome this limitation, we employed CASIN, a selective Cdc42 inhibitor (Fig. 2*E*). Treatment of both CD4^+^ SP thymocytes and peripheral CD4^+^ T cells with 10 µM CASIN did not affect surface TCRβ expression (Fig. 2*E*), but led to a marked reduction in surface microvilli and a modest decrease in cell size (Fig. 2 *F* and *G*). As a control, we examined whether CASIN treatment reduced the number and length of cell surface microvilli in MC38 epithelial cells (*SI Appendix*, Fig. S3*E*).

### T Cells Lacking or Inhibiting Cdc42 Show Attenuated TCR Signaling and Antigen Recognition on APCs.

**Fig. 3.**
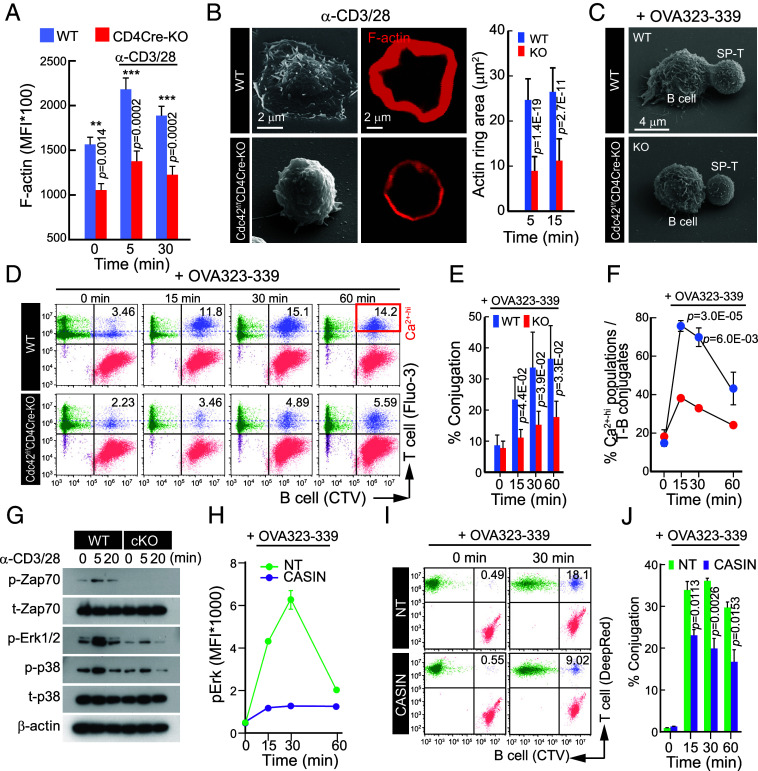
CD4^+^ SP thymocytes lacking Cdc42 show attenuated TCR signaling and antigen recognition on APCs. (*A*) Time-dependent changes in F-actin levels in CD4^+^ SP thymocytes from *Cdc42^f/f^* (WT) and *Cdc42^f/f^*CD4Cre (KO) following stimulation with soluble anti-CD3/CD28 antibodies. (*B*) Representative SEM and confocal images of cells stimulated on anti-CD3/CD28 antibody-coated glass coverslips for 5 min (n = 50 cells). (*C*) Representative SEM images showing conjugated cells. (*D*–*F*) Calcium influx and conjugation assay between CD4^+^ SP thymocytes and APCs. Conjugated cells were analyzed by flow cytometry (*D*) and quantified (*E* and *F*). (*G*) TCR proximal signaling (Zap70, Erk, p38) of CD4^+^ SP thymocytes from WT and KO mice on anti-CD3/CD28 Abs-coated glass at 5, 20 min. (*H*) ERK signaling with or without CASIN treatment. (*I* and *J*) Conjugation assay between CASIN-treated or untreated CD4^+^ SP thymocytes and APCs. Conjugated cell populations were analyzed by flow cytometry (*I*) and quantified (*J*). Data represent the mean ± SEM from three independent experiments. Statistics was performed using Student’s *t* test.

Given the reduced number and length of microvilli in Cdc42-deficient T cells (Fig. 2*A*) and their actin-based structure, we examined actin dynamics following TCR activation, primarily using SP thymocytes. Although still transitional, SP thymocytes have undergone positive selection and express high levels of TCRs, adhesion, and costimulatory molecules, enabling effective conjugate formation with APCs (22, 23). Thus, they serve as a suitable surrogate for peripheral T cells—especially in *Cdc42^f/f^* CD4-Cre mice, where peripheral CD4^+^ T cells are scarce or outnumbered by γδ T cells (*SI Appendix*, Fig. S2 *A* and *B*), making conventional analysis unreliable. CD4^+^ SP thymocytes from Cdc42-cKO mice exhibited normal actin responses to TCR stimulation but had significantly lower baseline F-actin levels, resulting in reduced overall F-actin accumulation (Fig. 3*A*). They also showed impaired spreading on anti-CD3/CD28-coated plates (Fig. 3*B*), indicating compromised actin dynamics and content. Similar defects were observed in peripheral CD4^+^ T cells from *Cdc42^f/f^* dLck-Cre mice (*SI Appendix*, Fig. S4 *A* and *B*).

The reduced number and length of actin-supported microvilli likely impair antigen recognition by CD4^+^ SP thymocytes (Fig. 3*C*), weakening their interaction with APCs and the associated calcium influx upon antigen stimulation (OVA323-339). To assess this, we evaluated conjugate formation and calcium signaling. CD4^+^ SP thymocytes were stained with Fluo-3, and B cells with CTV, allowing simultaneous monitoring of calcium influx in conjugates. Cdc42-deficient thymocytes showed significantly fewer conjugates (Fig. 3 *D*–*F*) and reduced Ca^2+^ influx (Fig. 3 *D* and *F*), along with decreased TCR-mediated signaling (Fig. 3*G*).

To address the challenge of reduced TCR expression on the surface of *Cdc42*-cKO T cells, we evaluated the effect of CASIN on TCR-mediated ERK signaling and OT-II CD4^+^ SP thymocyte–B cell conjugate formation. Notably, despite unchanged surface TCR levels, CASIN treatment significantly reduced phospho-ERK levels and impaired SP thymocyte–B cell conjugation (Fig. 3 *H*–*J*). In parallel, similar results were also obtained from peripheral CD4^+^ T cells obtained from OT-II mice (*SI Appendix*, Fig. S5 *A*–*C*). These findings suggest that Cdc42 contributes to the maintenance of microvilli, which in turn may facilitate effective TCR signaling and immune synapse formation—supporting a role for microvilli beyond simple antigen recognition.

### T Cells Lacking Cdc42 Display Fewer TCR Microclusters and a Diminished Release of Immunological Synaptosomes.

**Fig. 4.**
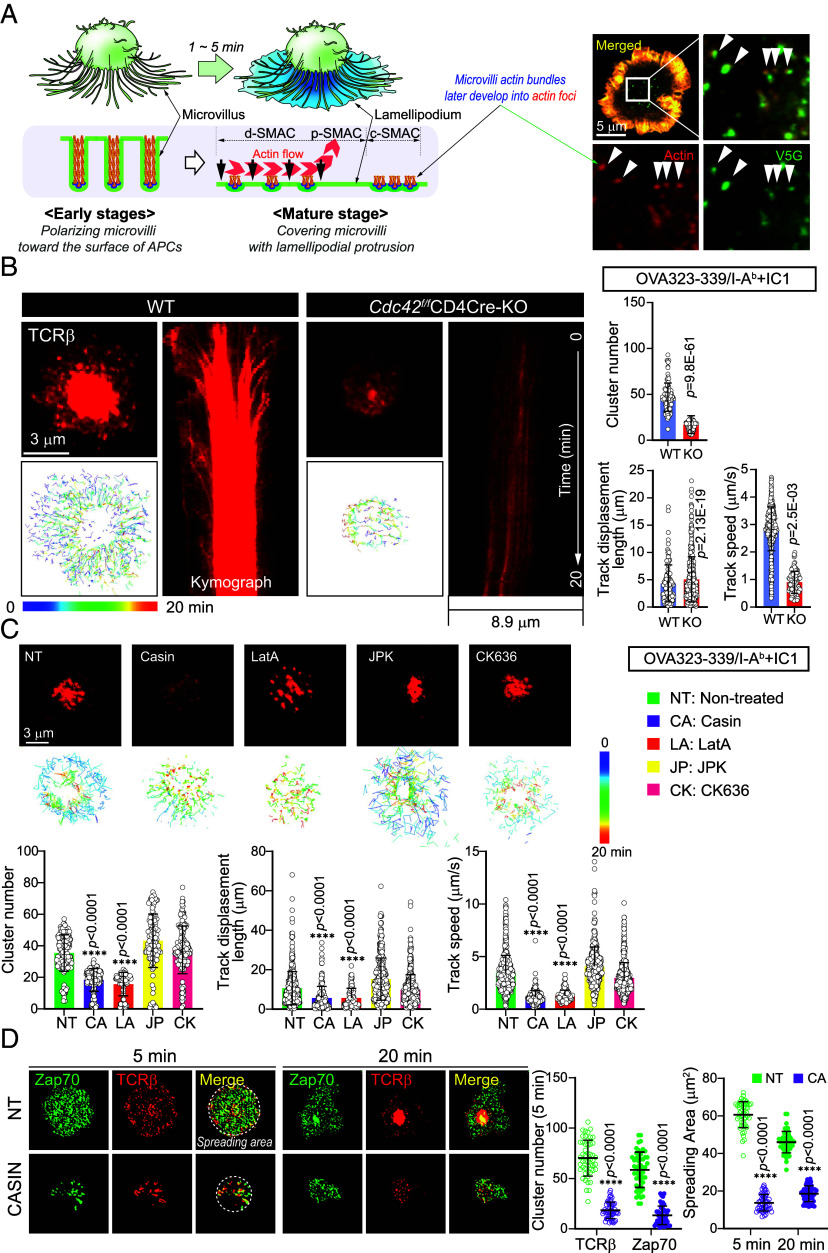
CD4^+^ SP thymocytes lacking Cdc42 exhibit reduced TCR microclusters. (*A*) Schematic diagram presenting a potential mechanism of how microvilli that disappear at the dSMAC move toward the cSMAC with TCR microclusters during immune synapse formation in T cells. The inset (*Right*) shows confocal microscopy images highlighting actin-foci (red) and V5G (green) in the central region of immune synapse, with the merged image (yellow) demonstrating the colocalization of actin and V5G (white arrows). (*B*) TIRF images showing TCR microclusters on a planar supported lipid bilayer (SLB) presenting OVA_323-339_/I-A^b^ and ICAM-1 (Movie S1). Individual TCR microclusters were tracked for 20 min. (*C*) Confocal microscopy images showing TCR microclusters on a SLB presenting OVA_323-339_/I-A^b^ and ICAM-1. OT-II CD4^+^ SP thymocytes were pretreated with various inhibitors and individual TCR microclusters were tracked for 20 min. Data represent the mean ± SEM of three independent experiments. (*D*) Confocal microscopy images showing TCRβ (red) and Zap70 (green) microclusters in OT-II CD4^+^ SP thymocytes (with or without CASIN) activated on SLB presenting OVA_323-339_/I-A^b^ and ICAM-1. Representative images are shown for each condition (*Left*). Microcluster numbers for TCRβ and Zap70 at 5 min (*Middle Left*), and cell spreading areas at both time points (*Right*), were quantified (n = 50 per group). Data were pooled from three independent experiments with randomly selected cells. Statistical analysis was performed using unpaired Student’s *t* test. ****P < 0.0001.

Based on previous studies, it was assumed that microvilli play a limited role in the formation and movement of TCR microclusters, as these structures were believed to collapse upon T cell activation (24). However, our research, along with others, has demonstrated that TCRs are enriched at the tips of microvilli (7, 8). These findings suggest that microvilli persist on T cells regardless of activation state and play a key role in directing TCR microclusters toward the c-SMAC. As shown in Fig. 1*B*, microvilli are not fully lost upon activation but are obscured by lamellipodia. Consistent with this, schematic and imaging data (Fig. 4*A*) indicate that dynamic microvilli movements help guide TCR microclusters. Notably, the microvilli-specific marker V5G colocalizes with actin foci, suggesting that microvilli actively regulate or restrict microcluster movement. At later stages, TCR clusters may even detach and transfer to the APC surface (Fig. 1*C* and *SI Appendix*, Fig. S6*A*). These observations challenge the notion that microvilli collapse during T cell activation and highlight their role in synapse organization.

We thus investigated how the reduction in the number and length of microvilli caused by *Cdc42*-knockout (*Cdc42^f/f^*CD4Cre OTII) affects the formation and movement of TCR microclusters. Furthermore, we explored the correlation between the release of microvilli and the shedding of TCR^+^ membrane particles. To confirm the clustering of TCRs, we utilized a supported planar lipid bilayer loaded with I-Ab MHC class II and ICAM-1. In *Cdc42*-KO SP thymocytes, the number of TCR microclusters decreased, and the formation of the c-SMAC was significantly reduced compared to WT SP thymocytes (Fig. 4*B* and Movie S1). The diminished formation of the c-SMAC indicates that Cdc42, which regulates microvilli, contributes to the spatial organization of TCR signaling during immune synapse formation. However, the fact that the track speed of the remaining TCR microclusters was not significantly affected suggests that the actin dynamics involved in membrane mobility are functioning normally in *Cdc42*-cKO SP thymocytes, as we observed in Fig. 3*A*. We also evaluated the effects of CASIN and other actin modulators on CD4+ thymocytes, including the G-actin polymerization inhibitor latrunculin A (LatA), the actin-stabilizing agent Jasplakinolide (JPK), and the Arp2/3 complex inhibitor CK636. Similar to the Cdc42 knockout, CASIN significantly reduced both TCR microcluster formation and c-SMAC assembly (Fig. 4*C*). LatA, which disrupts T cell microvilli (13), dramatically decreased TCR microclusters and reduced track speed (Fig. 4*C*). In contrast, JPK and CK636—agents that preserve microvilli integrity—had little effect on c-SMAC formation and microcluster dynamics (Fig. 4*C*), suggesting that intact microvilli are a prerequisite for proper TCR microcluster assembly and c-SMAC organization. Similar results were also observed in peripheral CD4^+^ T cells isolated from OT-II transgenic mice (*SI Appendix*, Fig. S6*B*).

CASIN has previously been reported to suppress T cell activation (25); however, whether this effect is linked to microvilli disruption has not been clearly established. Given that the tips of microvilli act as key sites where TCR-associated signaling molecules such as Zap70 are spatially organized, the integrity of these structures is critical for proper TCR signaling. We hypothesized that if CASIN disrupts microvilli, it may also impair the formation of TCR signaling microclusters. Supporting this, CASIN treatment not only led to a marked reduction in the number of TCR–Zap70 microclusters, but also impaired cell spreading (Fig. 4*D*)

**Fig. 5.**
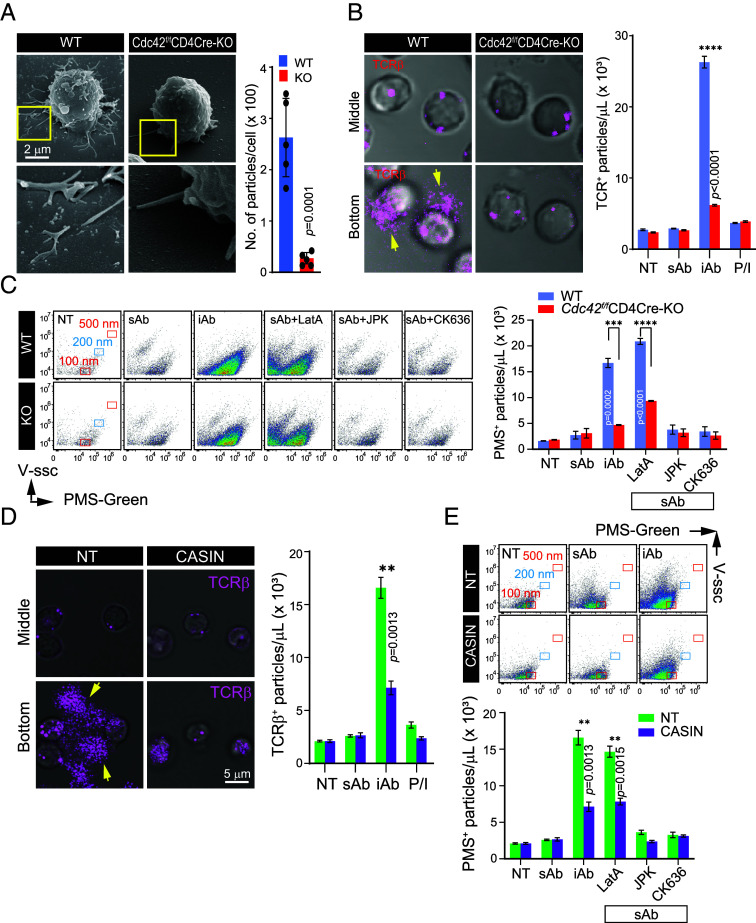
T cells lacking Cdc42 exhibit a normal machinery for vesicle formation but reduced immunological synaptosome generation. (*A* and *B*) SEM image (*A*) and confocal analysis, and side scatter-based FACS analysis (*B*) demonstrating the release of T cell microvilli particles. CD4^+^ SP thymocytes from *Cdc42^f/f^* (WT) and *Cdc42^f/f^*LckCre (KO) mice were placed on plated-coated anti-CD3/CD28 for 3 h, and then the cells were subjected for SEM and confocal analysis. (*C*) Flow cytometric quantification of extracellular vesicles from CD4^+^ SP thymocytes. Scatter plots (*Left*) show PMS-Green signal intensity in WT and KO cells under different conditions. Bar graph (*Right*) shows the number of PMS^+^ particles. (*D*) Confocal imaging and flow cytometric analysis of extracellular vesicles from CD4^+^ T cells with or without CASIN treatment. (*E*) Flow cytometry-based quantification of PMS^+^ particles. Scatter plots (*Top*) show PMS-Green intensity, and bar graph (*Bottom*) shows particle counts. Data are presented as mean ± SEM from three independent experiments. Statistical analysis was performed using Student’s *t* test.

As we presented in Fig. 1*C* and reported previously (7), the last step of T cell activation is the release of TIS mainly through shedding of TCR-enriched microvilli particles (TMPs) either when interacting with APCs, or when exposed to activation matrices such as lipid bilayers embedding p-MHC and ICAM-1, or anti-CD3/28 Abs-coated plates (7, 13). Dramatic reduction of TCR^+^ TIS release was observed in *Cdc42*-cKO SP thymocytes on plates coated with anti-CD3/28 Abs (Fig. 5*A*). Further, the amount of TCRβ^+^ fluorescent signals on the bottom was significantly reduced in *Cdc42*-cKO SP thymocytes (Fig. 5*B*), suggesting that Cdc42 contributes to the formation or release of microvilli-derived membrane particles from activated T cells. Our previous work showed that T cells release TMPs only when TCR activation is accompanied by adhesion signals like ICAM-1, and not by soluble anti-CD3/CD28 (sAb) alone (13). To investigate further, we compared vesicle release under three conditions: untreated (NT), sAb, and immobilized anti-CD3/CD28 (iAb). Consistent with earlier findings (7, 13), CD4^+^ SP thymocytes released almost no PMS^+^ vesicles under NT or sAb, but showed significant release under iAb stimulation (Fig. 5*C*). LatA enhanced PMS^+^ particle release under sAb, while other actin modulators had little effect. In contrast, Cdc42-cKO cells showed markedly reduced vesicle release (Fig. 5*C*).

Similarly, CASIN treatment reduced both TCR^+^ particles (Fig. 5*D*) and PMS^+^ vesicles (Fig. 5*E*) in peripheral CD4^+^ T cells, indicating that this is due to impaired membrane shedding rather than low TCR expression. These findings suggest that intact microvilli contribute to TIS biogenesis, with adhesion signals playing a particularly critical role in regulating their release, possibly in coordination with TCR engagement.

### *Cdc42^f/f^*CD4Cre-Knockout Slightly Upregulates Gene Clusters Related to Actin Cytoskeleton and Membrane Vesicle Formation.

**Fig. 6.**
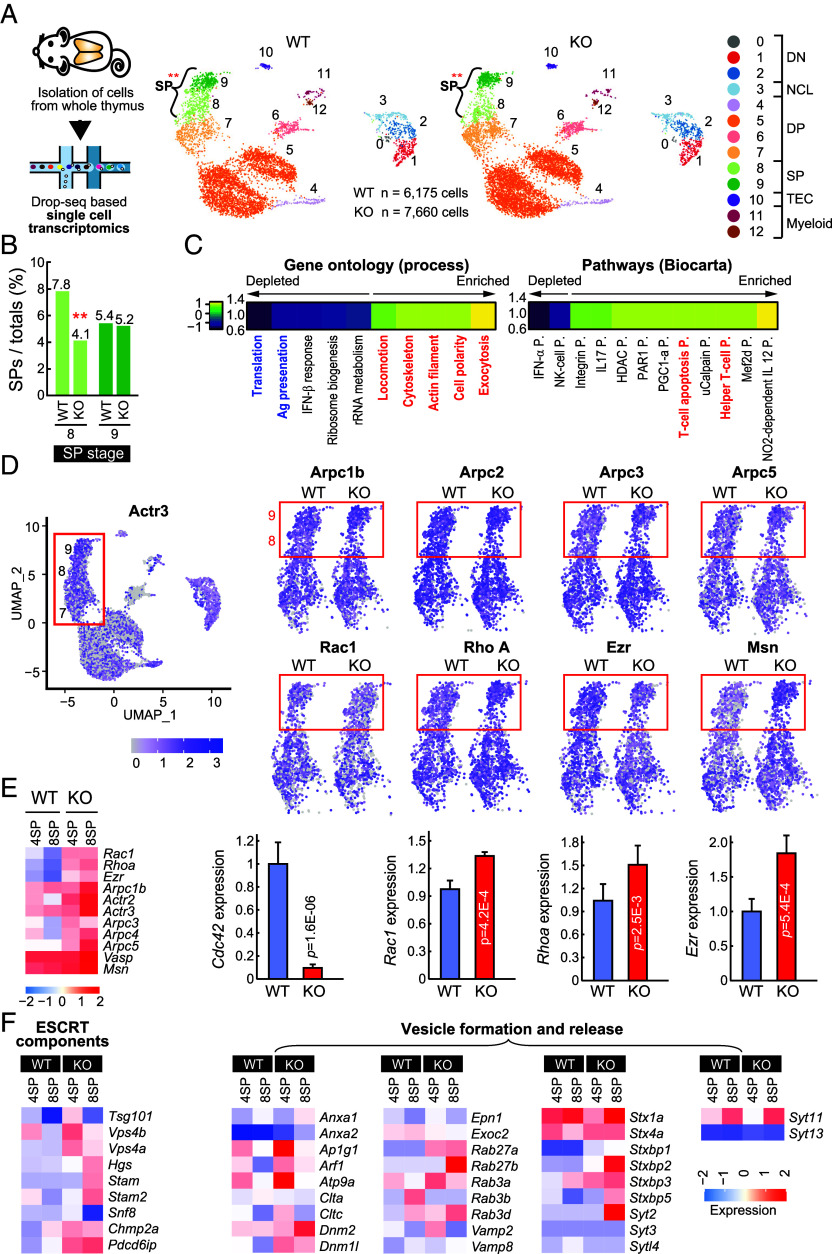
CD4^+^ SP thymocytes lacking Cdc42 slightly upregulate genes related to the actin dynamics and membrane vesicle formation. (*A*) Schematic diagram of scRNA-seq process and UMAP visualization of the cellular composition of *Cdc42^f/f^* (WT) and *Cdc42^f/f^*CD4Cre (KO) mice thymus. Five mice per group were killed for this experiment. 13 clusters were analyzed from the thymus and colored by cell types (DN, NCL, DP, SP, TEC, and Myeloid). (*B*) Bar graphs represent the cell populations of SP cells. Cluster 8 (SP stage) was significantly decreased in cell numbers among KO thymus (from 7.8% to 4.1%. Chi-square tests *P*-values < 0.01) (double red dots) (*C*) Gene ontology (GO) and pathway enrichment analysis of differentially expressed genes in cluster 8 and 9 of WT and Cdc42 KO thymocytes. (*D*) UMAP plots showing the expression levels of actin-related genes in WT and KO cells. The boxed areas highlight the changes in expression within the SP populations (clusters 8 and 9). (*E*) Heatmap of key genes involved in actin dynamics in SP thymocytes and real-time quantitative PCR analysis for the validation of actin-regulators (*Cdc42, Ezr, Rac1, and Rhoa*). Data represent the mean ± SEM of three independent experiments. (*F*) Heatmaps of the expression levels of genes associated with the ESCRT components and vesicle formation/release in SP thymocytes from WT and KO mice.

To understand the effects of the loss of *Cdc42* on the regulation of global genes and its connection to the formation of microvilli in thymocytes and peripheral T cells, we performed single-cell RNA sequencing (scRNA-seq) of whole thymus of WT and *Cdc42^f/f^*CD4Cre-knockout mice. After single-cell bioinformatics processing, 13,835 high-quality cells were identified and among these cells, 6,175 cells (44.6%) were obtained from WT condition and 7,660 cells (55.4%) from KO condition. Then, total thymic cells were classified into 13 clusters and each cluster’s cell type was annotated by the expression of specific marker genes (*SI Appendix*, Fig. S7 *A* and *B*). Of the 13 clusters, 10 were annotated as thymocytes, including 3 DN clusters, 1 nonconventional lymphoid cell (NCL) cluster, 4 DP clusters, and 2 SP clusters. There was also 1 thymic epithelial cell (TEC) cluster and 2 myeloid cell clusters. The number of cells were significantly decreased between WT and KO conditions in cluster 8 (chi-square tests *P*-values < 0.01), which was annotated as CD4 SP thymocytes (Fig. 6 *A* and *B*). Although both CD4^+^ and CD8^+^ SP thymocytes were reduced in *Cdc42* KO mice as shown by flow cytometry, only CD4^+^ SP cells showed a marked decrease in the scRNA-seq data, likely due to their higher basal frequency and more substantial reduction. As shown in Fig. 2*A*, single-cell analysis also revealed that CDC42 expression was lowest at the DP3 stage (*SI Appendix*, Fig. S7*C*).

GO enrichment analysis revealed that processes related to actin filaments, cytoskeleton, locomotion, and exocytosis were up-regulated in *Cdc42*-cKO mice (Hypergeometric tests *P*-values < 0.05) (Fig. 6*C*). The increased expression of actin-related genes is likely a compensatory response to the loss of Cdc42, but may also reflect activation of alternative cytoskeletal pathways, stress responses, or the selective survival of cells with intrinsically higher expression of these genes.

Pathway analysis also revealed an increased T cell apoptosis pathway in *Cdc42*-KO mice (Fig. 6*C*). Gene ontology analysis was used to investigate the expression of actin function-related proteins in SP thymocytes. Hierarchical clustering of gene expressions showed that genes involved in lamellipodia formation (*Rac1, Ezr, Msn, Arpc1, Arpc2, Arpc3,* and *Arpc5*) and stress fiber formation (*RhoA, Rock1, and Rock2*) were increased in *Cdc42*-KO mice (Fig. 6 *C* and *D*). The increased expression of actin regulators, such as *Ezrin*, *Rac1*, and *Rhoa*, was confirmed by quantitative RT-PCR (Fig. 6*E*).

Interestingly, although the release of TCR^+^ membrane particles (PMS^+^ vesicles) was significantly reduced in *Cdc42*-cKO cells compared to WT, the expression levels of genes related to ESCRT components, which are crucial for vesicle scission, and other factors involved in vesicle formation were either similar or even higher in the cKO cells (Fig. 6*F*). This discrepancy suggests that the machinery involved in vesicle formation and release, such as ESCRT-mediated pathways, remains functional or even upregulated in Cdc42-deficient cells. However, despite intact ESCRT machinery, the reduced release of TCR^+^ or PMS^+^ membrane vesicles indicates that proper microvillar architecture is also essential for vesicle shedding from the plasma membrane. This highlights a critical upstream role for Cdc42 in maintaining microvilli integrity. Furthermore, the formation of these membrane vesicles relies heavily on adhesion-dependent signaling pathways, which in turn require the structural integrity of microvilli. To identify key regulators of cell state changes by *Cdc42*-KO, we performed regulon analysis to identify transcription factors of given cell clusters using PROGENy algorithm (26) and the DoRothEA dataset (27), a database of interactions between transcription factors and their target genes (regulons). These resources were combined to identify important regulons and pathway activity in each cluster. In cluster 8, where microvilli in T cells were defective, approximately 10 regulons were significantly increased (*SI Appendix*, Fig. S8*A*). Some of these regulons, such as *Stat4, Irf1*, and *Nfkb2,* were significantly decreased in *Cdc42*-KO mice (*SI Appendix*, Fig. S8*B*). These transcription factors are known to play a role in the development of CD4 and CD8 T cells (28–30), suggesting that Cdc42 plays a crucial role in the transition from the DP to SP stage.

We also observed a decrease in the gene expression of CCR7 and L-selectin (Sell) (*SI Appendix*, Fig. S8*C*), both of which are well known to localize at the tips of microvilli (31, 32). Consistent with reduced mRNA expression, surface CCR7 levels were significantly decreased in Cdc42-cKO thymocytes (*SI Appendix*, Fig. S8*D*). Correspondingly, SP thymocytes from these mice showed poor spreading and impaired migration on ICAM-1-coated surfaces in response to CCL19 (*SI Appendix*, Fig. S8*E*). These defects suggest impaired CCR7 signaling, possibly due to disrupted microvilli localization, which may contribute to reduced cell adhesion.

### T Cells Lacking Cdc42 Exhibit a Severe Defect in Adhesion to High Endothelial Venules (HEV) and APCs In Vivo, without Affecting Their Migratory Properties.

**Fig. 7.**
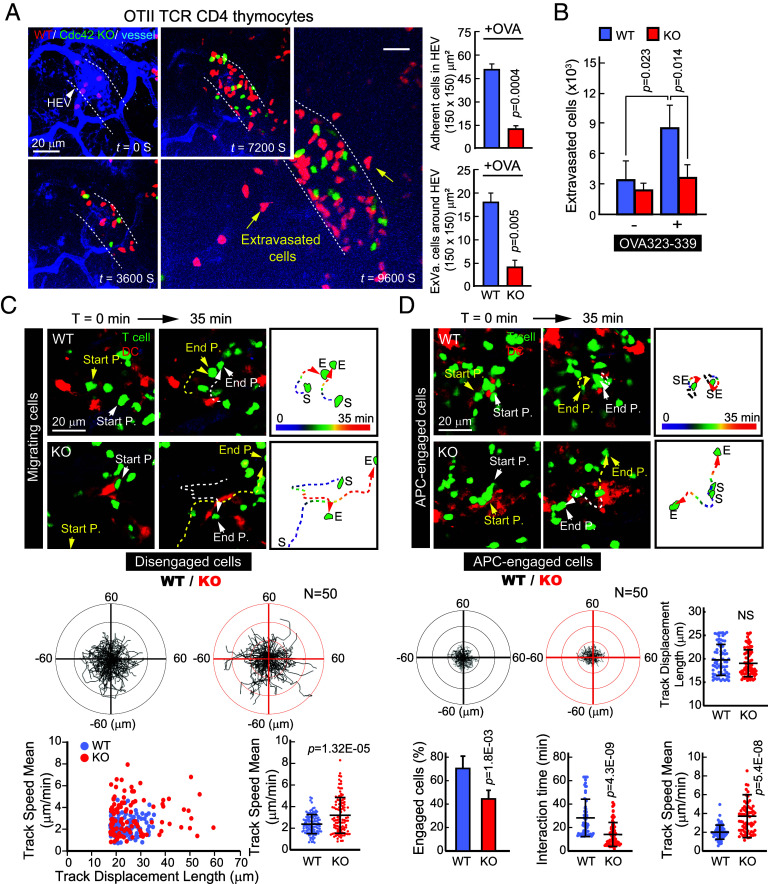
CD4^+^ SP thymocytes lacking Cdc42 exhibit a severe defect in adhesion to HEV and APCs in vivo, without affecting their migratory properties. (*A*) Representative snap shots of in vivo imaging of HEV binding and extravasation at the indicated time points. The popliteal lymph node was imaged for 3 h and the number of HEV-bound or extravasated T cells were analyzed at 9600s. (Movie S2). (*B*) Homing of CD4^+^ thymocytes into the popliteal lymph node in the presence or absence of OVA_323-339_-pulsed DCs. Data represent the mean ± SEM of three independent experiments (*C* and *D*) Representative snap shots of in vivo imaging of OT-II CD4^+^ SP thymocytes (1 × 10^7^) randomly migrating in the presence of OVA_323-339_-pulsed DCs (*C*) or interacting with OVA_323-339_-pulsed DCs (*D*) (5 × 10^6^). Tracks of representative two cells were displayed as white and yellow dot line. Arrowheads indicate start (S) and end (E) point of each track. (Movies S3 and S4). Trajectories of migrating cells or APC-engaged cells are displayed. Track displacement and track speed mean were analyzed in randomly selected migrating cells. The % of APC-engaged cells, interaction time, track displacement length, and track speed mean were analyzed in randomly selected APC-engaged cells (N = 50). The dot plot dataset (*C* and *D*) represents the sum of results obtained from three independent experiments in which equal cells were randomly selected in each experiment. Results are representative of three independent experiments. Statistical significance was determined using unpaired Student’s *t* test.

To determine whether defects in cell adhesion and antigen sensing are reproducible in vivo, we extended our work to living mice. As recent thymic emigrants, CD4^+^ SP thymocytes migrate through lymph nodes and transiently express adhesion molecules such as LFA-1 and ICAM-1, allowing them to physiologically engage with APCs in these tissues (33). To this end, we used *Cdc42^f^*^/^*^f^*CD4Cre OTII mice that recognize the OVA323-339 peptide and performed three-dimensional live imaging of lymph nodes using two-photon microscopy. DCs pulsed or not pulsed with OVA323-339 were injected 48 h prior to the experiment, followed by intravenous injection of OTII CD4 SP thymocytes. Fig. 7*A* (Movie S2) shows that OTII CD4 SP thymocytes from *Cdc42*-cKO mice (shown in green) did not adhere to HEVs in lymph nodes as effectively as CD4 SP thymocytes from WT mice (shown in red). As a result, a dramatic reduction in extravasation was observed in *Cdc42*-cKO OTII CD4 SP thymocytes (Fig. 7*B*). These results suggest that Cdc42 deficiency impairs T cell adhesion to HEVs, consequently hindering their extravasation through HEVs.

We next investigated whether Cdc42-deficient CD4^+^ SP thymocytes exhibit reduced motility, as previously observed in vitro (*SI Appendix*, Fig. S4*E*). Given their elevated expression of lamellipodia- and stress fiber–associated proteins, we hypothesized that migration might be preserved despite Cdc42 deficiency. To test this, we tracked OTII CD4^+^ SP thymocytes in lymph nodes. Unlike WT cells, which accumulated and maintained interactions with DCs, Cdc42-deficient cells failed to engage DCs and rapidly disappeared (Movies S3 and S4).

To characterize their behavior, we classified cells as either migratory or APC-engaged. In the migratory group, Cdc42-deficient cells traveled longer distances, with increased track displacement and faster speeds compared to WT cells (Fig. 7*C*), indicating ineffective APC targeting. In the APC-engaged group, WT thymocytes formed stable contacts with DCs, while Cdc42-deficient cells showed reduced engagement, shorter interaction duration, and faster motility (Fig. 7*D*). Although displacement during engagement was similar, the overall proportion of engaged Cdc42-deficient cells was significantly lower. A comparable pattern was also observed in CASIN-treated peripheral CD4^+^ T cells, consistent with the response seen in CD4^+^ SP thymocytes (*SI Appendix*, Fig. S10 and Movie S5).

Taken together, these results indicate that Cdc42 deficiency in CD4 SP thymocytes leads to impaired adhesion ability, which follows weakened adhesion signals. Despite this, the overall migratory capacity of Cdc42-deficient thymocytes was not negatively affected, as they exhibited increased movement speed and displacement compared to WT cells.

## Discussion

T cell microvilli, slender projections originating from the plasma membrane of T cells, serve as the primary loci for antigen recognition and signal initiation (6, 34). These structures share a structural similarity with filopodia in that they both consist of a core of actin filaments enveloped by actin-binding proteins, and play a role in cell motility (4, 5). However, the molecular composition of T cell microvilli distinguishes it from filopodia, as it contains specialized machinery for antigen recognition and signaling, whereas filopodia contain a more generic set of actin-binding proteins (34). Despite their importance, the regulation of T cell microvilli during development remains poorly understood. Our study highlights the critical role of Cdc42 in microvilli formation during the DP-to-SP transition. By positioning TCRs at their tips, microvilli contribute not only to TCR microcluster formation but also to their movement toward the c-SMAC, promoting stable immunological synapse formation. In Cdc42-cKO or CASIN-treated T cells, reduced microvilli number and length disrupt microcluster organization and impair T cell activation, underscoring the structural and functional importance of microvilli in TCR signaling.

T cell development in the thymus involves a series of morphological changes essential for their function (35, 36). While these changes have drawn increasing interest, it remains unclear how microvilli are regulated during this process. Notably, DP thymocytes exhibit fewer microvilli and smaller size, whereas more mature SP thymocytes display higher microvilli density. This may partly explain differences in TCR expression between the stages. Given their functional maturity, SP thymocytes likely require more microvilli for efficient antigen recognition, which is critical for central tolerance by eliminating autoreactive cells and promoting regulatory T cell development (37). Interestingly, in *Cdc42*-cKO thymocytes, TCR expression is markedly reduced, mimicking the characteristics observed in DP thymocytes. This suggests that *Cdc42* deficiency may hinder the developmental progression of thymocytes, maintaining them in a less mature state with fewer microvilli and lower TCR expression, much like DP thymocytes. However, it remains unclear whether Cdc42 regulates microvilli dynamics and TCR expression independently or if these processes are coregulated throughout different stages of T cell development (20, 38–40). Notably, the observation that CASIN disrupts microvilli without altering surface TCR expression highlights the critical role of microvilli integrity in sustaining TCR-Zap70 signaling strength and facilitating effective antigen recognition by T cells. While it remains possible that other factors contribute to the effects of Cdc42 loss, the current findings underscore Cdc42-mediated microvilli regulation as an important determinant of effective antigen recognition and immune responses.

Although Cdc42 is a key regulator of actin polymerization, its deletion in Cdc42-cKO cells triggers compensatory upregulation of actin-related proteins such as Rac1, RhoA, and ERM family members (37). This may help maintain actin dynamics and cell migration. However, these changes might also reflect activation of alternative cytoskeletal pathways, stress responses, or selective survival of cells with high basal expression, making interpretation difficult. Interestingly, inhibition of the Arp2/3 complex by CK636—acting downstream of Rac1—did not affect TCR microcluster formation or T cell activation, suggesting Rac1 is not essential in this context. While Rac1 typically promotes lamellipodia, its overexpression in Cdc42-deficient thymocytes may be disruptive, as prior studies show Rac1 activity can shorten T cell microvilli (24). This may contribute to the observed microvillar reduction, though our data show microvilli are shortened, not lost (Fig. 4*A*), and still support TCR microcluster assembly and signaling. Overall, Cdc42 plays a nonredundant role in organizing actin-rich microvilli critical for T cell activation, while Rac1 appears to have a limited or even antagonistic role.

Investigating Cdc42 function in T cells using conditional knockout models posed several challenges. In CD4-Cre mice, peripheral CD4^+^ and CD8^+^ T cells were nearly absent, likely due to developmental or survival defects, making mature T cell analysis unfeasible. In dLck-Cre mice, deletion occurred later but showed variable Cdc42 expression in peripheral T cells, limiting reproducibility. To overcome this, we focused on CD4^+^ SP thymocytes, where Cdc42 deletion was more consistent and microvilli are well developed during the DP-to-SP transition—coinciding with increased TCR, adhesion, and costimulatory molecule expression (41). This population is well established for studying TCR signaling and actin remodeling during thymocyte maturation (42, 43). We also included peripheral CD4^+^ T cells from dLck-Cre mice to confirm broader relevance. The consistent findings across both cell types highlight the limitations of current knockout models and underscore the need for refined tools. Future studies using inducible systems like CreERT2 may enable more precise Cdc42 deletion in peripheral T cells while preserving thymic development.

Despite retaining overall migratory capacity, Cdc42-deficient T cells—including SP thymocytes—show impaired ability to form stable interactions with DCs and establish functional immunological synapses, highlighting a specific role for Cdc42 in microvilli-mediated T cell–APC engagement. While actin remodeling for motility appears preserved, likely through compensatory pathways, it is insufficient to support microvilli-dependent functions such as sustained adhesion and signaling. These cells also display increased motility within lymphoid tissues, likely due to reduced APC dwell time or impaired integrin engagement. Their inability to adhere stably to HEVs and APCs suggests that Cdc42 is essential not only for migration but also for organizing membrane protrusions critical for immune activation. Thus, the reduced TCR signaling observed is best explained by microvilli defects rather than global actin disruption.

Earlier models suggested that TCR microclusters form through random interactions of freely mobile membrane proteins (10–12). However, recent studies have shown that microclusters are spatially confined to microvilli-rich contact sites between T cells and APCs [4, 7], highlighting a structural role for microvilli in organizing and guiding TCR clusters (4, 7). This has introduced a new perspective on TCR microcluster formation, where microvilli play a structural role, organizing and guiding these clusters (7, 8). In Cdc42-cKO or CASIN-treated T cells, we observed reduced TCR microclusters and impaired migration toward the c-SMAC, suggesting that microvilli not only cluster TCRs but also help direct their transport. Although previous reports indicated that microvilli collapse upon T cell activation via Rac1 (24), our data suggest they persist at the periphery beneath lamellipodia (Fig. 1*B*), continuing to concentrate TCRs at their tips (Fig. 1*C*). These microvilli may be carried inward by retrograde actin flow. Since TIRF microscopy captures only two-dimensional movement, the observed TCR cluster motion likely reflects the movement of microvilli themselves. A marked reduction in TCR clusters after LatA treatment further underscores the importance of microvilli in TCR microcluster formation and dynamics. In contrast, Arp2/3 seems dispensable, as TCRs are localized to microvilli tips rather than lamellipodia.

Previously, we demonstrated that TCR microclusters reaching the c-SMAC are shed as microvilli-derived particles, potentially delivering signaling components to APCs (7). Interestingly, this shedding is closely linked to T cell proliferation (13). In Cdc42-cKO T cells, TCR^+^ particle release is significantly reduced compared to WT cells, correlating with decreased proliferation. This reduction is not due to lower TCR expression, as CASIN-treated cells show similar decreases in TCR^+^ particles. This is supported by an overall decline in PMS^+^ vesicles in both Cdc42-cKO and CASIN-treated cells. Despite the absence of Cdc42, genes related to exocytosis and the ESCRT pathway remain unaffected or even upregulated, suggesting intact conventional vesicle secretion machinery.

In summary, our study shows that microvilli are essential, dynamically regulated structures during T cell development. Cdc42 is required for their formation in mature SP thymocytes, and loss of microvilli disrupts chemokine receptor localization, TCR positioning, migration, and antigen recognition. These defects impair TCR signaling toward the c-SMAC, potentially leading to anergy and reduced clonal expansion. Although TCR^+^ microvilli particle production is reduced in Cdc42-cKO T cells, exocytosis and ESCRT pathways remain intact, indicating that impaired signaling, not secretion, is the primary issue. Future studies should investigate how Cdc42 and actin regulators control microvilli dynamics and T cell function.

## Materials and Methods

### Mice, Cells, and Chemical Treatment.

*Cdc42^f/f^* transgenic, CD4Cre transgenic, and *OTII* TCR transgenic mice were purchased from the Jackson Laboratory (Bar Harbor, ME). *Cdc42^f/f^* mice were crossed with CD4Cre and dLckCre mice to generate lineage specific knockout mice, respectively. *Cdc42^f/f^* and *Cdc42^f/f^* CD4Cre mice were further crossed with OTII to generate an OVA-specific TCR transgenic line. All mice were housed in specific pathogen-free conditions. CD4^+^ SP thymocytes and peripheral CD4^+^ T cells were isolated by MojoSort™ Mouse CD4 Selection Kit (BioLegend, San Diego, CA). For CASIN experiments, CD4 T cells or CD4 SP thymocytes were pretreated with CASIN (10 µM) for 2 h and stimulated in cell culture medium containing CASIN or DMSO (NT). All experiments are performed as the mean ± SEM (or SD) from at least three independent experiments and were analyzed using Student’s *t* test (*P* < 0.05). Scanning electron microscopy was performed at the GIST Advanced Institute of Instrumental Analysis. A list of cells and reagents used in this study can be found in *SI Appendix* and All other detailed methods are described in *SI Appendix*.

## Supplementary Material

Appendix 01 (PDF)

Movie S1. **TCR clustering of CD4^+^ SP thymocytes in the supported lipid bilayer**. CD4^+^ SP thymocytes obtained from the thymus of OTII crossed *Cdc42^f/f^* and *Cdc42^f/f^*CD4Cre mice were stained with anti-TCRβ (H57Fab-Alexa594) and stimulated on supported lipid bilayer with OVA_323-339_/I-A_b_ and ICAM-1. Centripetal movement of TCR microclusters was imaged for 20 min every 12s using a TIRF microscope. This video corresponds to Figure 4B.

Movie S2. **CD4^+^ SP thymocytes from Cdc42 KO mice exhibit severe defect in adhesion to HEVs *in vivo***. Two-photon intravital imaging of CD4^+^ SP thymocytes at the HEVs of the popliteal lymph node. CD4^+^ SP thymocytes from *Cdc42^f/f^* OT-II (WT) and *Cdc42^f/f^* CD4-Cre OT-II (KO) mice were labeled with CMRA (orange) and CMFDA (green), respectively, and intravenously injected into WT recipient mice. The recipients had been injected 24 h earlier with OVA_323-339_-pulsed BMDCs. HEVs were visualized by intravenous injection of Dextran-Cascade Blue™. The popliteal lymph node was imaged using two-photon microscopy for 3 h. This video corresponds to Figure 7A.

Movie S3. **Cdc42-KO CD4^+^ SP thymocytes move faster than wild-type CD4^+^ SP thymocytes at the popliteal lymph node (Site I)**. Two-photon microscope imaging of CD4^+^ SP thymocytes at popliteal lymph node. CMRA-labelled and OVA_323-339_-pulsed BMDCs were injected to footpad of recipient wild-type mice. CMFDA-labelled CD4^+^ SP thymocytes (OTII crossed *Cdc42^f/f^*CD4Cre) were i.v. injected at 24 h post DC injection and popliteal lymph node was imaged for 2 h at 24 h post injection of CD4^+^ SP thymocytes. Two migrating cells (noninteracting cells) were randomly selected in each condition, highlighted as white and red dot circle, and tracked for 35 min. A track path was visualized as dot line. This video corresponds to Figure 7C.

Movie S4. **Reduced interaction of CD4^+^ SP thymocytes from Cdc42 KO mouse with antigen-pulsed DCs at the popliteal lymph node (Site II)**. Two-photon microscope imaging of CD4^+^ SP thymocytes interacting with antigen-pulsed DCs at popliteal lymph node. CMRA-labelled and OVA_323-339_-pulsed BMDCs were injected to footpad of recipient wild-type mice. CMFDA-labelled CD4^+^ SP thymocytes (OTII crossed *Cdc42^f/f^*CD4Cre) were i.v. injected at 24 h post DC injection and popliteal lymph node was imaged for 2 h at 24 h post injection of CD4^+^ SP thymocytes. Two DC-interacting T cells were randomly selected in each condition, highlighted as white and red dot circle, and tracked for 35 min. A track path was visualized as dot line. This video corresponds to Figure 7D.

Movie S5. CASIN-treated OTII CD4^+^ T cells exhibit a severe defect in adhesion to HEVs in vivo. OTII CD4^+^ T cells, pretreated with or without CASIN, were labeled with CMRA (orange) or CMFDA (green), respectively, and intravenously injected into wild-type recipient mice that had received _323-339_-pulsed BMDCs 24 h earlier. HEVs were visualized using Dextran–Cascade Blue™. The popliteal lymph node was imaged by two-photon microscopy up to 3000s. This video corresponds to Figure S10.

## Data Availability

scRNAseq data have been deposited in GEO (NCBI) (GSE289949) (44). All other data are included in the manuscript and/or supporting information.
