# Supplementary material for: Cdc42 defect reveals insights into microvilli organization and function in T cell immunity
Source: Proc Natl Acad Sci U S A. 2025 Jul 25;122(30):e2505291122. doi: 10.1073/pnas.2505291122 (PMC12318239; doi:10.1073/pnas.2505291122)
Supplement: Supplementary file 1 — Appendix 01 (PDF) [file pnas.2505291122.sapp.pdf]

Supporting Information for

Cdc42 defect reveals insights into microvilli organization and function in T-cell immunity

Won-Chang Soh[1,2], Sang-Moo Park[1,2], Jeong-Su Park[1,2], Hatice Karabulut[1,2], Hee-Tae Kang[1,2], Sun-Kyoung Kang[1,2], Min-Sang Kim[1,2], Jihwan Park[1], Sun-jae Lee[1], Hye-Ran Kim[3*], and Chang-Duk Jun[1,2*]

*Correspondence and requests for materials:
Dr. Hye-Ran Kim, Division of Rare and Refractory Cancer, Tumor Immunology, Research Institute, National Cancer Center, 323 Ilsan-ro, Ilsandong-gu, Goyang-si Gyeonggi-do, Korea. Tel: +82-31-920-2462; E-mail: hrkim@ncc.re.kr
Dr. Chang-Duk Jun, School of Life Sciences, GIST, 123 Cheomdangwagi-ro, Gwangju 61005, Korea. Tel: +82-62-715-2506; Fax: +82-62-715-2546; E-mail: cdjun@gist.ac.kr

This PDF file includes:

Supporting text
Figure legends S1 to S10
Legends for Movies S1 to S5
SI References

Other supporting materials for this manuscript include the following:

Movies S1 to S5

Supporting Information Text

Materials and Methods

Reagents and antibodies

Antibodies against mouse TCRβ (MABF931), tetramethylrhodamine (TRITC)-phalloidin, Poly-L-lysine (PLL), 50% glutaraldehyde, and osmium tetroxide were purchased from Sigma-Aldrich (St. Louis, MO, USA). Antibodies against Cdc42, RhoA, and Rac1 were purchased from Abcam (Cambridge, MA, USA). Antibodies against β-actin (cst8457), anti-rabbit IgG-HRP (cst7074), anti-phospho-p44/42 MAPK (33370), anti-Zap70 (2705S), anti-phospho-Zap70 (2701S), anti-p38 MAPK (9212S), anti-phospho-p38 MAPK (9215 S), and anti-mouse IgG-HRP (cst7076) were purchased from Cell Signaling Technology (Danvers, MA, USA). Anti-CD2 antibody (130-100-617) was purchased from Miltenyi Biotec (Bergisch Gladbach, Germany). Fluorescence-conjugated anti-TCRβ (109212), TCRγδ (107503), CD3ε (152304), CD4 (100509), CD8 (100711), CD25 (16210325), CD69 (104511), LFA-1 (141005), TRIM (627203), and CCR7 (12010550) were purchased from BioLegend (San. Diego, CA, USA). Hybridoma cell lines for anti-CD3ε (145-2C11; CRL-1975) and anti-CD28 (PV1; HB-12352) were purchased from the American Type Culture Collection (Manassas, VA, USA). Anti-rabbit IgG-Alexa Fluor™ 488, Fluo-3/AM, 7-AAD and Total exosome isolation Reagent were purchased from Invitrogen (Carlsbad, CA, USA). Anti-TCRβ (H57-597) was purchased from Bio-X-Cell (West Lebanon, NH, USA). TRITC-labeling kit, CellTracker™ Green CMFDA, Orange CMRA, CellTrace™ Violet, annexin V- PE, Alexa Fluor™ 594 labeling kit, and Fab preparation kit were purchased from Thermo Fisher Scientific (Waltham, MA, USA). Recombinant mouse ICAM-1, mouse IL-2 DuoSet ELISA, mouse IFN-γ DuoSet ELISA, and Substrate Reagent Pack (DY999) were purchased from R&D Systems (Minneapolis, MN, USA). MHC I-Ab was provided by the NIH Tetramer Core Facility (Atlanta, GA, USA). WEST-ZOL western blot detection kit was purchased from Intron Biotechnology (Seongnam, Korea). Complete protease inhibitors and phosphatase inhibitors were purchased from Roche Applied Science (Indianapolis, IN, USA). OVA peptide fragments (323–339 and 257–264) were purchased from GeneScript (San Francisco, CA, USA). CASIN was purchased from Sanbio BV (Uden, North-Brabant, Netherlands)

Cells

$CD4^+$ SP thymocytes were isolated from the mouse thymus by MojoSort™ Mouse CD4 Selection Kit (BioLegend, San Diego, CA, USA). Peripheral CD3 and CD4 T cells were isolated from the mouse spleen and lymph nodes by MojoSort™ Mouse CD3 and CD4 Selection Kit (BioLegend, San Diego, CA, USA). To generate mouse T-cell blasts, Peripheral CD3 and CD4 T cells were stimulated on 2 μg/ml anti-CD3/CD28 Abs-coated culture plates with 100 U/ml rIL-2 for 48 h and cultured for 5 days with 100 U/ml rIL-2. For CASIN experiments, CD4 T cells or CD4 SP thymocytes were pre-treated with CASIN (10 μM) for 2 h and stimulated in cell culture medium containing CASIN or DMSO (NT). Naïve B cells were isolated from the spleen by EasySep™ Mouse B Cell Isolation Kit (Stemcell Technologies, Vancouver, Canada). To generate B cell blasts, naïve B cells were cultured for 3 days in complete RPMI containing LPS (10 μg/mL). Bone marrow was flushed from femur and tibia bones, and bone marrow-derived DCs (BMDCs) were grown with the addition of 20 ng/mL recombinant murine granulocyte-macrophage colony-stimulating factor (GM-CSF) for 8 days. GM-CSF was added at every 3 days. MC38 (ENH204-FP) cells were purchased from Kerafest (Boston, MA).

Animals

Cdc42$^{f/f}$ transgenic, CD4Cre transgenic, *OTII* TCR transgenic mice were purchased from the Jackson Laboratory (Bar Harbor, ME, USA). *Cdc42$^{f/f}$* mice were crossed with CD4Cre and dLckCre mice to generate lineage specific knockout mice, respectively. *Cdc42$^{f/f}$* and *Cdc42$^{f/f}$* CD4Cre mice were further crossed with OTII to generate an OVA-specific TCR transgenic line. All mice were housed in specific pathogen-free conditions. All experimental methods and protocols were approved by the Institutional Animal Care and Use Committee of the School of Life

Sciences, Gwangju Institute of Science and Technology (Gwangju, Korea), and carried out in accordance with their approved guidelines (IACUC GIST-2018-066 and GIST-2021-091).

Western blot

Cells were lysed in ice-cold lysis buffer (50 mM Tris-HCl pH 7.4, containing 150 mM NaCl, 1% Triton X-100, 1 tablet of complete protease inhibitors, and phosphatase inhibitors) for 30 min on ice. Lysates were centrifuged at 16,000 × g for 30 min at 4°C, and the supernatant was mixed with sodium dodecyl sulfate (SDS) sample buffer (100 mM Tris-HCl pH 6.8, 4% SDS, 20% glycerol, bromophenol blue) and heated for 5 min. The proteins were separated through 12% SDS-PAGE gels and were transferred to a nitrocellulose membrane by means of a Trans-Blot SD semidry transfer cell (Bio-Rad, California, USA). The membrane was blocked in 5% skim milk for 1 h, washed, and incubated with primary antibodies in TBS containing 0.1% Tween 20 (TBS-T) and 5% skim milk overnight. Excess primary antibody was removed by washing the membrane four times in TBS-T. And then the membrane was incubated with peroxidase-labeled secondary antibody (0.1 μg/ml) for 1.5 h. Bands were visualized with a WEST-ZOL Western Blot Detection kit (Intron Biotechnology) and exposed to X-ray film.

Single-cell RNA seq

Total thymocytes were prepared and analyzed by the single-cell RNA sequencing as described previously (45). Using R Seurat package from the output of Cell Ranger software, single-cell sequencing raw data was processed and analyzed. In brief, low-quality cells were removed if (1) number of genes per cell< 200 or > 6,000; and (2) number of reads per cell > 50,000. Regulon activity analysis was performed by combining of R *progeny* and *dorothea* packages for WT and KO conditions (28, 46). Pathway enrichment analysis were performed to search for enriched biological processes of given cell cluster and conditions by R *fgsea* package (p-values < 0.05) and normalized enrichment scores of given pathways were visualized by heatmaps.

Reverse transcription PCR (RT-PCR) and real-time quantitative RT-PCR (qRT-PCR)

Total RNA was isolated from cells or homogenized tissues of C57BL/6 mice with TRIzol reagents (Molecular Research Center, Cincinnati, OH, USA) and reverse transcribed using RT-Premix (Intron Biotechnology). PCR was performed with the respective forward and reverse primers; mouse *Gapdh*, 5′- CATCACTGCCACCCAGAAGACTG-3′ and 5′-ATGCCAGTGAGCTTCCCGTTCAGGGTGAA-3′; mouse *Rpl13a*, 5′-GCTGCTCTCAAGGTTGTTCG-3′ and 5′- TCTGCCTGTTTCCGTAACCT-3′; mouse *Cdc42*, 5′-GTGTGTTGTTGTTGGTGATGGT-3′ and 5′-CAGTGGTGAGTTATCTCAGGCA-3′; mouse *Rac1*, 5′- CCGCAGACAGACGTGTTCTTAA-3′ and 5′- AGCTTCGTCAAACACTGTCTTG-3′; mouse *Rhoa*, 5′-TGGTGATGGAGCTTGTGGTAAG-3′ and 5′-GGCACATTTGGACAGAAATGCT-3′; mouse *Ezr*, 5′-GACAAGAAGGCACCTGACTTTG-3′ and 5′-CCTCTTGGTCTTCTGCTCGTAG-3′; mouse *Ccr7*, 5′-GACACGCTGAGATGCTCACT-3′ and 5′-GTTGAGCTGCTTGCTGGTTT-3′; The expression levels of mouse Cdc42, Rac1, RhoA, Ezrin and CCR7 were evaluated by qRT-PCR. Amplification was performed in a StepOne real-time PCR system (Applied Biosystems, Norwalk, CT, USA) for continuous fluorescence detection in a total volume of 10 μL of cDNA/control and gene-specific primers using SYBR Premix Ex Taq (TaKaRa Bio). The mRNA levels of the target genes were normalized relative to those of *Gapdh* using the following formula: relative mRNA expression = $2^{-(\Delta Ct \text{ of target gene} - \Delta Ct \text{ of Gapdh})}$, where Ct is the threshold cycle value. In each sample, the expression of the analyzed gene was normalized to that of *Gapdh* and described as the mRNA level relative to *Gapdh.*

Flow cytometry

Cells were stained with florescent-conjugated antibodies for 30 min at 4℃. For intracellular staining, cells were permeabilized using Cytofix/Cytoperm (Invitrogen) and stained with the antibody for 30 min at 4℃. Data were acquired on a FACSCanto (BD Biosciences, San Jose, CA) and analyzed with FlowJo software (Tree Star, Ashland, OR).

Intracellular staining of phosphorylated protein by flowcytometry

To measure the expression of phospho-Erk1/2 from activated T cells, CD4 T cells and CD4 thymocytes were activated on anti-CD3/CD28 (10 and 2 μg/ml) Abs-coated culture plates for 0, 15, 30 and 60 min with CASIN or not. The cells were then fixed for 30 min with 2% paraformaldehyde (PFA) and permeabilized with 100% methanol overnight at -20℃. After permeabilization, the cells were incubated with APC-conjugated phospho-Erk1/2 at 4℃ for 1 hour, followed by washing and analyzed by flow cytometry.

Ca^{2+} influx and conjugation assay

CD4 SP thymocytes from *Cdc42^f/f^* and *Cdc42^f/f^* CD4Cre mice were labeled with 4 μg/ml Fluo-3/AM (Invitrogen) for 1 h at 37°C, washed, and resuspended with cold PBS containing of biotinylated anti-CD3 (5 μg/ml; 145-2C11) and anti-CD28 (5 μg/ml; 37.51). Cells were incubated for 30 min at 4°C and further incubated for 20 min at RT. To crosslink antibodies, streptavidin (25 μg/ml) was immediately added before flow cytometry. Data were analyzed with FlowJo software (TreeStar, Ashland, OR, USA).

To measure conjugation and Ca^{2+} influx together, CD4 SP thymocytes from $Cdc42^{f/f}$ and $Cdc42^{f/f}$ CD4Cre OTII mice were stained with 4 μg/ml Fluo-3/AM and B cell blasts pulsed with 1 μg/ml $OVA_{323-339}$ peptides were stained with CellTrace™ Violet. To measure conjugation in CASIN experiments, CD4 T cells and thymocytes from OTII mice were stained with 1 μg/ml DeepRed and B cell blasts pulsed with 1 μg/ml $OVA_{323-339}$ peptides were stained with CellTrace™ Violet. T cells were incubated with B cell (1:1 ratio) for 0, 15, 30 and 60 min. The relative proportion of conjugated cells and $Ca^{2+\ high}$ populations were determined by FACS Canto and analyzed with FlowJo software.

Cell transfection and viral infection

For retroviral transduction, $CD4^+$ T cells from WT and OTII TCR transgenic mice were activated on plates coated with 2 μg/mL anti-CD3/28 in the presence of 100 U/mL rIL-2 for 48 h. Retroviral particles were produced either by co-transfecting V5G_pMSCV or TCRζ_tdTomato_pMSCV with the pCL-Eco packaging vector or by individually transfecting each construct into packaging cells using Lipofectamine 2000 (Invitrogen, Carlsbad, CA). After 48 h, viral supernatants were collected and mixed with 1 × 10^6 mouse T cells, which were then plated on 12-well plates pre-coated with 20 μg/mL RetroNectin (Clontech, Mountain View, CA). Cells were spin-infected at 2000 × g for 90 min at 25°C in the presence of 100 U/mL rIL-2. Post-infection, the T cells were maintained in fresh mouse T-cell media supplemented with rIL-2 and expanded for an additional 3 days. For transient transfection, Jurkat T cells were transfected with V5G_N1 using either Lipofectamine 2000 or Amaxa Nucleofector II.

Confocal analysis

To measure the expression and movement of V5G and TCR ζ_tomato signals from activated T cells, transfected Jurkat cells and mouse CD4 blasts were activated on anti-CD3/CD28 antibodies (10 and 2 μg/ml) for various time points (basal, 5, 15, 30, 60 min). For cell spreading analysis, CD4 SP thymocytes from $Cdc42^{f/f}$ and $Cdc42^{f/f}$CD4Cre mice were plated on coverslips coated with 10 and 2 μg/ml anti-CD3/CD28 antibodies for 5, 15, and 30 min. The cells were then fixed for 10 min with 4% paraformaldehyde (PFA) and permeabilized with 0.1% Triton-X (Sigma-Aldrich) in PBS for 10 minutes at room temperature (RT). After permeabilization, A). To screen for SMAC formation during T cell activation, OT-II T cells were pretreated with actin modulators, such as Lat A (237 nM), JPK (100 nM), CK 636 (100 μM) and CASIN (10 μM), for 30 min before imaging and maintained in the presence of these modulators throughout imaging. The supported planar lipid bilayer was prepared as described previously (8). For TCR and Zap70 microcluster imaging, OT-II $CD4^+$ single-positive thymocytes were pretreated with CASIN (10 μM) for 2 h at 37°C, then transferred onto planar supported lipid bilayers presenting $OVA_{323-339}/I\text{-}A^b$ and ICAM-1, and allowed to interact for either 5 or 20 min. Cells were subsequently fixed with 4% paraformaldehyde for 10 min, permeabilized with 0.1% Triton X-100 for 5 min, and blocked with 1% BSA. They were then incubated overnight with primary antibodies against TCRβ (AF594-conjugated) and Zap70, followed by 2 h incubation with Alexa Fluor 488-conjugated anti-rabbit IgG secondary antibodies, and washed thoroughly. For Imaging was performed using a FV-1000

or FV-3000 confocal microscope (Olympus, Tokyo, Japan) or a Laser Scanning Microscopy VIS Laser module, LSM 880 (Carl Zeiss, Germany). Microvilli intensity and cell actin ring areas were analyzed using ImageJ software (NIH, Bethesda, MD)

Quantification of T-cell immunological synaptosomes (TISs)
The number of $TCR\beta^+$ membrane particles (by SEM) around one single cell were analyzed by ImageJ software (NIH, Bethesda, MD). Six major steps were applied as follows: (1) background subtraction, (2) sharpening and finding edges, (3) smoothing and converting to black and white, (4) closing and filling holes, (5) denoising and segmenting, and (6) filtering and measuring.

Chemokinesis assay
Freshly isolated CD4 SP thymocytes from *Cdc42$^{f/f}$* and *Cdc42$^{f/f}$* CD4Cre mice were placed on 10 µg/ml of rICAM-1 coated plate. CCL19 (final concentration: 50 nM) was treated and cells were immediately imaged for 20 min by EVOS FL Auto 2 (Invitrogen, Carlsbad, CA). To measure cell polarity, moving distance, and number of adherent cells, the images were analyzed using ImageJ software.

TIRF microscopy
The supported planar lipid bilayer was prepared as described previously (1). CD4 SP thymocytes from *Cdc42$^{f/f}$* and *Cdc42$^{f/f}$* CD4Cre OTII mice and peripheral $CD4^+$ T cells from WT C57BL/6N mice were stained with anti-TCRβ (H57Fab-Alexa594) for 1 h at 4°C. Cells were washed with cold PBS and resuspended with HEPES buffered saline. Cells were placed on supported lipid bilayer with $OVA_{323-339}$/I-Ab and ICAM-1 and immediately imaged for 20 min by TIRFM (IX-81; Olympus, Tokyo, Japan) equipped with a solid-state laser (543 nm, 20 mW; Coherent, Santa Clara, CA).

In vivo homing assay
CD4 SP thymocytes from *Cdc42$^{f/f}$* and *Cdc42$^{f/f}$* CD4Cre mice were stained with CMFDA or CMRA, respectively. Cells were then injected intravenously and mice were sacrificed at 4 h post injection. For isolation of lymphocytes from organ tissues and blood, mice were euthanized, and organs and blood were prepared. Lungs and livers were minced into small fragments and digested with 1 mg/ml collagenase D (Sigma) and 50 mg/mL DNase (Roche) at 37℃ for 45 min, passed through a 70 µm cell strainer, and lymphocytes were isolated via Percoll gradient centrifugation. LNs and spleens were made single cells through 40 µm cell strainers. RBCs in single cells suspensions were lysed with ACK lysis buffer (Thermo), washed with DPBS and prepared for flow cytometry.

Two-photon imaging
To evaluate CD4 SP thymocyte migration, $OVA_{323-339}$ - pulsed BMDCs were stained with CMRA-Orange and injected to footpad of recipient wild-type C57BL/6 mice (5 x 10^6). CD4 SP thymocytes from *Cdc42$^{f/f}$* and *Cdc42$^{f/f}$* CD4Cre OTII T cells (1 x 10^7) were stained with CMFDA-Green and i.v. injected at 24 h post injection of DCs. On the next day, recipient mice were anesthetized using Isofluorane and i.v. injected 200 µL of Dextran-Cascade Blue™. Popliteal lymph node of recipient mice was imaged for 3 h using Zeiss LSM 880 microscope equipped with a MaiTai laser (Coherent) tuned to 780 nm in combination with an NDD2 BIG2 GaAsP detector and a 20 × water-dipping lens (NA 1.0, Zeiss) using ZEN v2.1 acquisition software. To examine T-cell extravasation, $OVA_{323-339}$ -pulsed BMDCs (5 x 10^6) were injected into the footpad of recipient mice. To assess the effect of CASIN on T-cell homing, OT-II $CD4^+$ T cells (1 x 10^7) were either treated with 10 µM CASIN for 2 h or left untreated, labeled with CMFDA or CMRA, respectively. Cells were intravenously injected into recipient mice 24 h after DC administration. Time-lapse imaging of the popliteal lymph node was performed using two-photon microscopy for up to 3000 seconds following T-cell injection.

Scanning electron microscopy (SEM)
DP thymocytes, CD4 SP thymocytes, naïve CD4 T cells, MC38 epithelial cells (with or without CASIN [10 µM] treatment), $CD4^+$ blasts, and B cell blasts were seeded on poly-L-lysine (PLL)-

coated silica plates, incubated for 30 min, and fixed overnight at 4°C in 2.5% glutaraldehyde in 0.1 M phosphate buffer. For activation, CD4 SP thymocytes and $CD4^+$ blasts were plated on silica surfaces coated with anti-CD3/CD28 antibodies and stimulated for 2 h before fixation under the same conditions. All samples were dehydrated through a graded ethanol series for 30 min, dried using a critical point dryer, coated with platinum by sputtering, and imaged using a focused ion beam scanning electron microscope (FIB-SEM; Hitachi, Tokyo, Japan) and ultra high resolution field emission scanning electron microscope (Verios 5 UC, Thermo Fisher Scientific, Waltham, MA, USA).

Quantification of extracellular vesicles (EVs)

$CD4^+$ SP thymocytes were stained with CellMask Green plasma membrane stain or anti-mouse TCRβ-AF488 for 30 min at 37°C. After staining, cells were washed twice by cold PBS and activated on iAb for 3 h at 37°C in plain RPMI medium. For inhibitor experiments, actin inhibitors (JPK (100 nM), CK636 (100 µM), and LatA (237 nM)) were applied after sAb (10 µg/ml) treatment. EVs were isolated using the Total Exosome Isolation Reagent following the manufacturer's protocol. Isolated EVs were then stained again with CellMask Green for 30 min in the dark at room temperature to ensure clear identification. The EV pellets were suspended in 1 mL of PBS for flow cytometric analysis. For CASIN experiments, CD4 T cells were pretreated with CASIN (10 µM) for 2 h and then stained with CellMask Green plasma membrane stain. After staining, the subsequent procedures were performed as described above.

Tumor animal models

B16F10 melanoma cells ($2×10^6$ cells) were s.c. injected into the two dorsal flank regions of *Cdc42*$^{f/f}$ and *Cdc42*$^{f/f}$CD4Cre mice (8-week old). Mice were sacrificed at day 21 post-inoculation of tumor cells. At the end of the experiments, tumors were isolated, weighed, and photographed for gross images. Tumor volume was measured using calipers every 3 days from the time T cells were injected and was calculated based on the formula: length × width × height (mm^3).

Image analysis

The imaging data, including the SEM, EVOS and confocal analysis were analyzed by ImageJ software. IMARIS software (BitPlane, Belfast, Northern Ireland) was used for Two-photon, TIRFM and confocal analysis. For TIRFM and confocal analysis, TCR cluster movements were tracked automatically using an algorithm of Brownian movement. For two-photon analysis, T cells were automatically tracked using an algorithm of Brownian movement and T cells were categorized into two groups: migratory cells and APC-engaged cells, based on the track displacement of T cells over a period of 2 h. The number of T cells and % of DC-engaged T cells, track speed, displacement, and duration were also analyzed using Imaris software.

Histological analysis

Thymuses were fixed in 10% PFA and washed to remove the PFA with three 10-min washes in PBS, dehydrated through a graded ethanol series, and embedded in paraffin. Sections were cut to 10 mm-thick, and mounted on glass slides (Leica) and left overnight in an incubator at 37 °C. They were then de-waxed in xylene and rehydrated in a graded series of ethanol baths, and stained with hematoxylin and eosin (HE). Whole thymus images were acquired using EVOS FL Auto 2 (Invitrogen, Carlsbad, CA, USA). The cortex-medullar ratio was calculated as following: ((total thymic area – thymic medullar area)/medullar area).

Statistics

Mean values were calculated using data taken from at least three independent experiments conducted on different days. Where significance testing was performed, student's t test. Differences between groups were considered significant at P-value < 0.05.

Figure S1. *Cdc42*$^{f/f}$CD4Cre-knockout results in impaired thymic T cell development

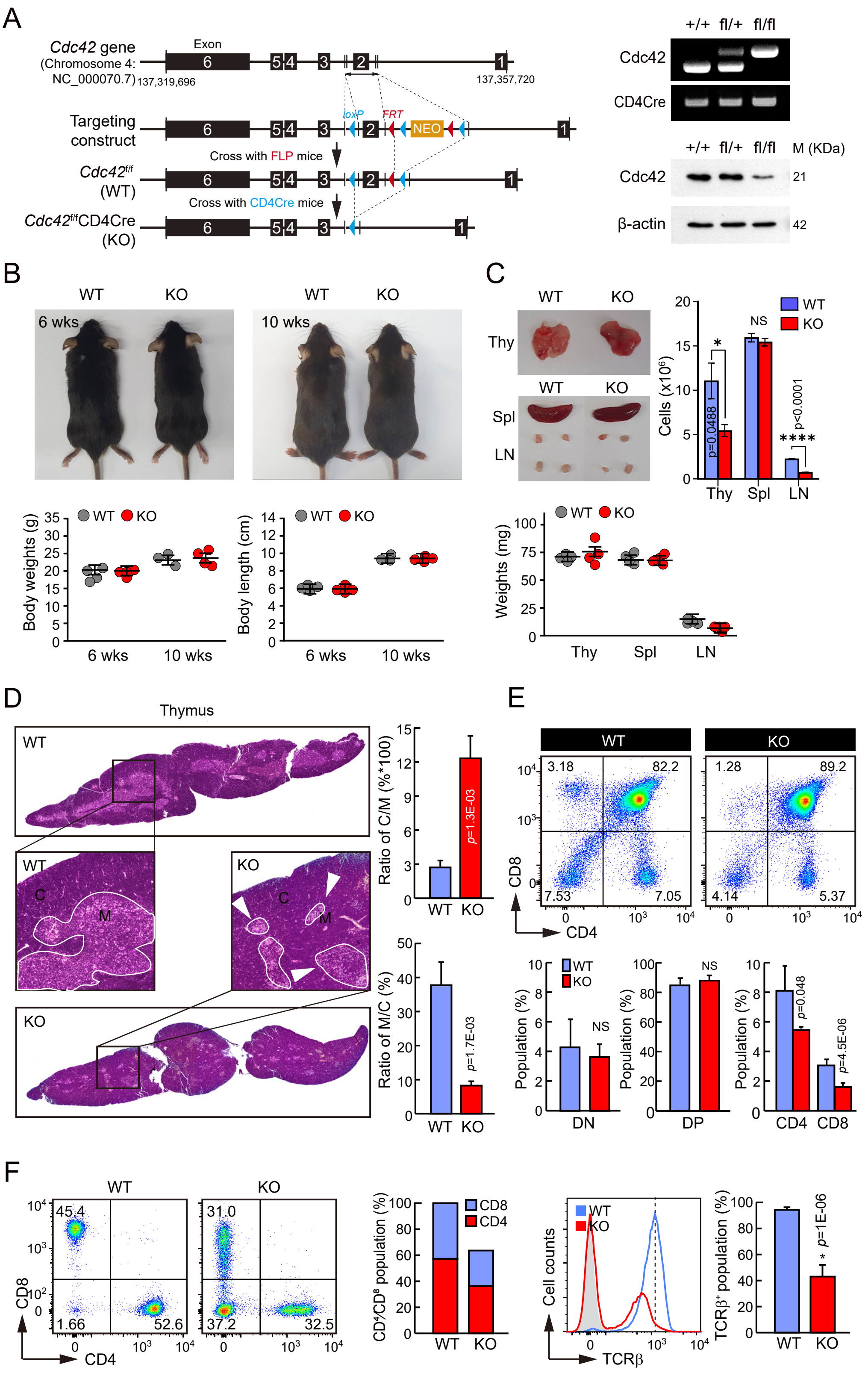

Figure S1. *Cdc42$^{f/f}$*CD4Cre mice results in impaired thymic T-cell development. (A) Schematic of the *Cdc42* floxed allele and breeding strategy for T cell-specific deletion. LoxP sites flank exon 2, and the NEO cassette was removed by *Flp* recombinase. CD4Cre-mediated recombination generates *Cdc42$^{f/f}$*CD4Cre mice. Representative genotyping PCR and immunoblot confirming Cdc42 deletion in CD4$^+$ SP thymocytes are shown. (B and C) Representative photograph of mouse, thymus, spleen, and lymph nodes of WT littermate control (n = 5) and Cdc42 KO (n = 5) mice at 6- or 10-week-old. The body weight and total length of mice are depicted below (A). Statistical analysis of thymus, spleen, and lymph node weight is shown in scatter graph (B). Thy, thymus; Spl, spleen; LN, lymph node. (D) Representative images of H&E staining of thymus (6-week-old mice) sections from WT and KO mice. White arrows heads indicate abnormal structure of medulla in KO mice. Ratio of cortical to medullary or medullary to cortical surface area in WT and *Cdc42*-cKO mice (N=10 sections from 3 thymi of 8 weeks old. (E) Flow cytometry analysis of *Cdc42$^{f/f}$* and *Cdc42$^{f/f}$*CD4Cre Thymic T cells. Each graph shows DN, DP, SP ratio. (F) Flow cytometry analysis of peripheral T cells from *Cdc42$^{f/f}$* and *Cdc42$^{f/f}$*CD4Cre. T cells were analyzed for CD4, CD8, and TCRβ expression. Data represent the mean ± SEM of three independent experiments. Statistics was performed using student's t test.

Figure S2. *Cdc42*$^{f/f}$CD4Cre-knockout mice show strong anti-cancer phenotype

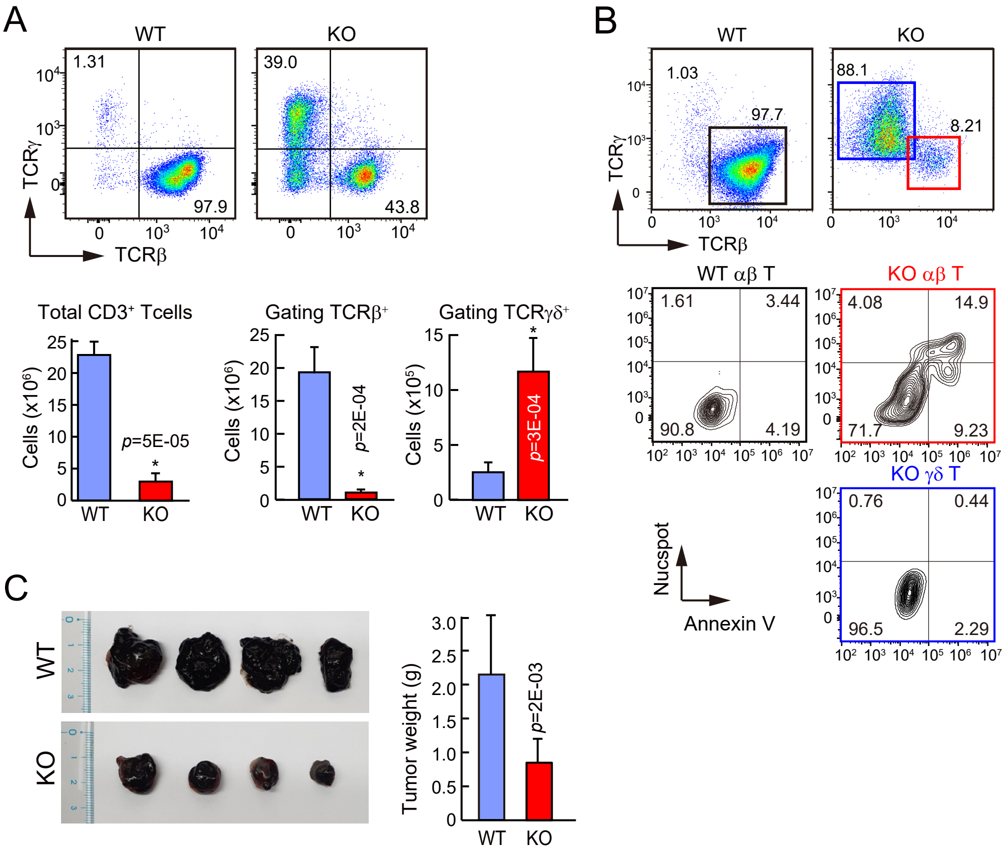

Figure S2. ***Cdc42$^{f/f}$*CD4Cre-knockout mice show strong anti-cancer phenotype.** (A) Flow cytometry analysis of peripheral T cells from *Cdc42$^{f/f}$* and *Cdc42$^{f/f}$*CD4Cre. T cells were analyzed for TCRγ and TCRβ expression with absolute cell numbers. (B) Analysis of TCR expression and apoptosis in *Cdc42$^{f/f}$* and *Cdc42$^{f/f}$* CD4Cre peripheral T cells. Representative flow cytometry plots showing TCRβ and TCRγ expression in *Cdc42$^{f/f}$* and *Cdc42$^{f/f}$* CD4Cre peripheral T cells (top). CD4Cre-mediated deletion of Cdc42 leads to a reduction in αβ T cells and relative increase in γδ T cells. Annexin V and Nucspot staining of WT αβ T cells, KO αβ T cells (red gate), and KO γδ T cells (blue gate) is shown (bottom), indicating increased apoptosis in KO αβ T cells. (C) B16F10 tumor growth of WT and KO mice (left). Tumor weights were significantly lower in KO mice compared to WT controls (right, p = 0.002; unpaired two-tailed t-test). Data are shown as mean ± SEM.

Figure S3. *Cdc42*$^{f/f}$dLckCre-knockout shows normal thymic T cell development

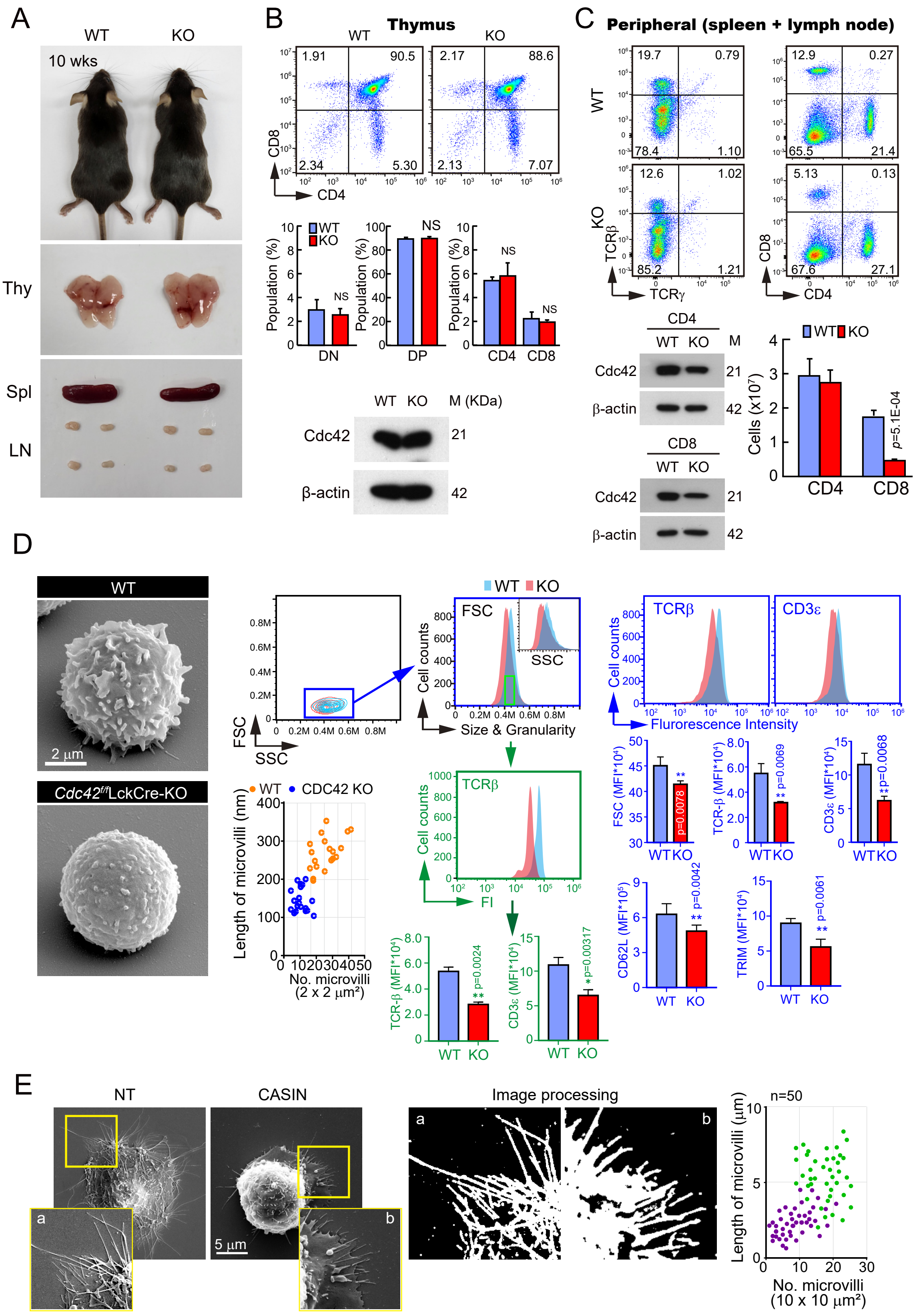

Figure S3. *Cdc42$^{f/f}$* dLckCre mice shows normal thymic T-cell development. (A) Representative pictures of mouse, thymus, spleen, and lymph nodes of *Cdc42$^{f/f}$* and *Cdc42$^{f/f}$*dLckCre mice at 10-week-old. (B) Representative flow cytometry plots of CD4 and CD8 stained thymocytes and quantification of the average percentages of DN, DP, CD4^{+} or CD8^{+} SP thymocytes obtained from Cdc42$^{f/f}$ and Cdc42$^{f/f}$dLckCre mice. (C) Flow cytometry analysis of $\alpha\beta$ T or $\gamma\delta$ T cells and CD4 or CD8 populations in spleen and lymph node. The number of CD4^{+} and CD8^{+} T cells was quantitated. (D) SEM images of peripheral CD4^{+} T cells from *Cdc42$^{f/f}$* (WT) and *Cdc42$^{f/f}$*LckCre (KO) mice (left). Flow cytometry analysis measuring FSC and SSC in T cells, along with the microvilli-associated proteins TCRβ and CD3ε (right). The data represent the mean ± SEM from three independent experiments. Data represent the mean ± SEM of three independent experiments. Statistics was performed using student's t test. (E) SEM images of non-treated (NT) and CASIN-treated MC38 cells (left). Yellow insets show magnified views of filopodia. Quantification of filopodia length and number per 10 × 10 μm^2 area from 50 images is shown (right).

Figure S4. CD4⁺ T cells from *Cdc42*$^{f/f}$dLck-Cre mice exhibit attenuated actin dynamics following TCR stimulation

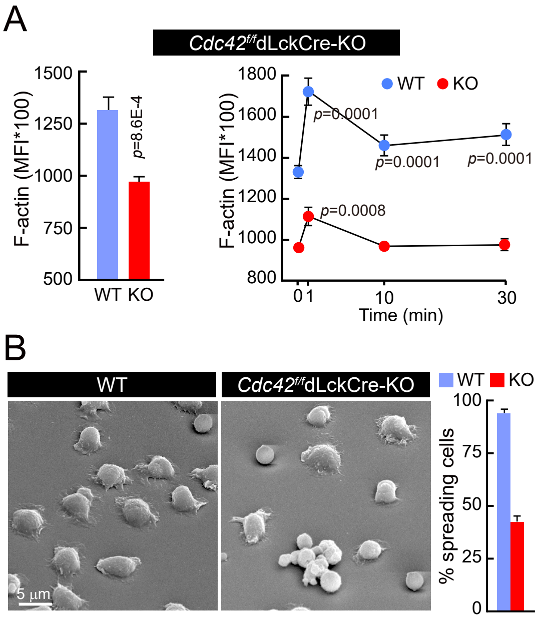

Figure S4. CD4$^+$ T cells from *Cdc42$^{f/f}$*dLck-Cre mice exhibit attenuated actin dynamics following TCR stimulation. (A) A representative histogram of the time-dependent F-actin contents of *Cdc42$^{f/f}$* and *Cdc42$^{f/f}$*dLck-Cre peripheral CD4$^+$ T cells. Cells were stimulated with soluble anti-CD3/CD28 Abs for the indicated time points, fixed, and stained with TRITC-phalloidin. Bar graph shows F-actin content in the resting condition. Data represent the mean ± SEM of three independent experiments. (B) Representative SEM images of peripheral CD4$^+$ T cells from *Cdc42$^{f/f}$* and *Cdc42$^{f/f}$* dLckCre mice on anti-CD3/CD28 Abs-coated glass for 5 min. At least 100 cells on random images were counted for statistical analysis on cell spreading. Data represent the mean ± SEM of three independent experiments.

Figure S5. OTII CD4⁺ T cells inhibiting Cdc42 show attenuated TCR signaling and antigen recognition on APCs

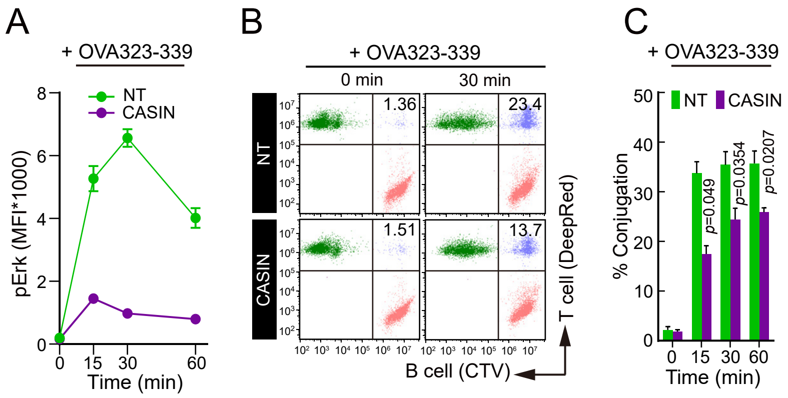

Figure S5. OTII CD4$^+$ T cells inhibiting show attenuated TCR signaling and antigen recognition on APCs. (A) ERK signaling in CD4$^+$ T cells with or without CASIN. CD4$^+$ T cells were pretreated with 10 μM CASIN for 2 h prior to the experiment. (B-C) Conjugation assay between CD4$^+$ T cells with or without CASIN and APCs. CD4+ T cells were stained with Cell Tracker Deepred and co-cultured with B cells, which were either pulsed with $OVA_{323-339}$ or not, for different durations (0 min, 15 min, 30 min, and 60 min). B cells were stained with CTV to distinguish them from T cells. Conjugated cells were analyzed by flow cytometry (B) and quantified (C). CD4$^+$ T cells were pretreated with 10 μM CASIN for 2 h prior to the experiment. Data represent the mean ± SEM of three independent experiments.

Figure S6. T cells lacking Cdc42 exhibit reduced TCR microclusters

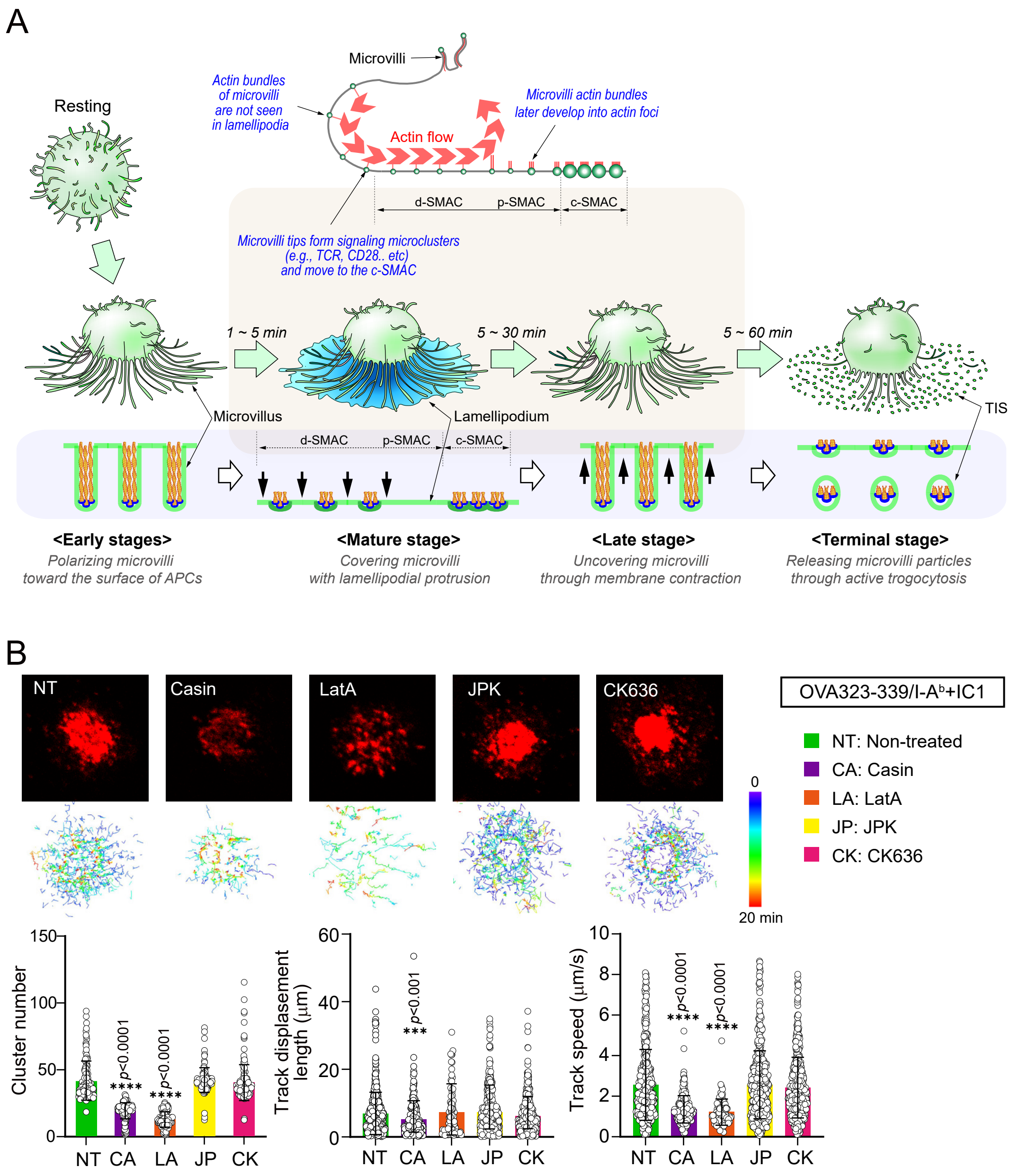

Figure S6. T cells lacking cdc42 exhibit reduced TCR microclusters (A) Schematic diagram presenting a potential mechanism of how microvilli that disappear at the dSMAC move toward the cSMAC with TCR microclusters during immune synapse formation, and reappear after immune synapse deformation in T cells. (B) Confocal microscopy images showing TCR microclusters on a planar bilayer presenting $OVA_{323\text{-}339}$/I-A^b and ICAM-1. OT-II $CD4^+$ T cells were pre-treated with CASIN (10 μM), LatA (237 nM), JPK (100 nM), or CK636 (100 μM) for 30 min, stained with anti-TCRβ (H57Fab-Alexa594), and individual TCR microclusters were tracked for 20 min. Data represent the mean ± SEM of three independent experiments. Statistics was performed using student's t test.

Figure S7. scRNA-seq analysis of total thymocytes

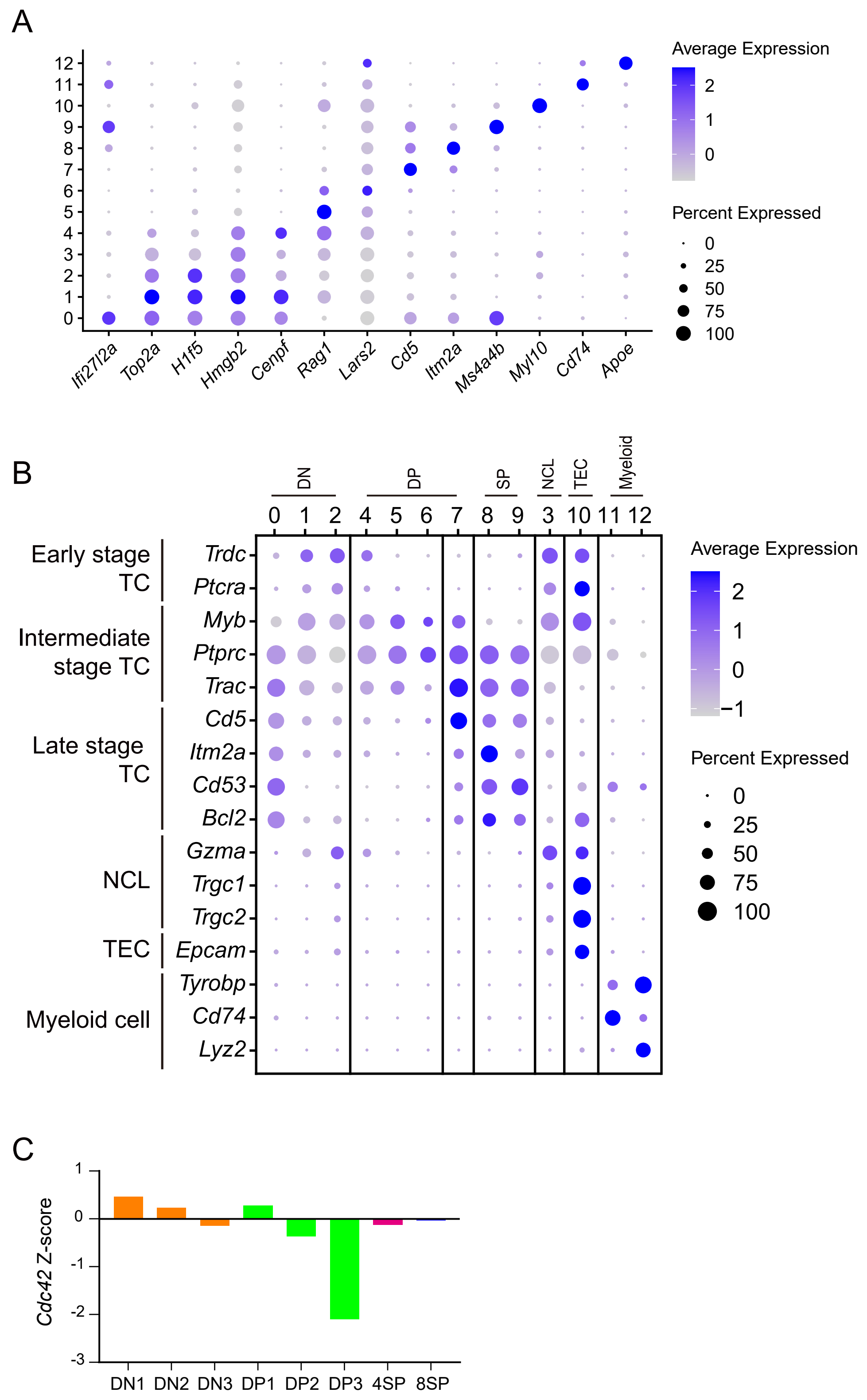

Figure S7. scRNA-seq analysis of total thymocytes. (A) Dot plot showing the expression of selected markers of 13 clusters. (B) Dot plot for expression of marker genes in different cell types. Color represents the average expression level for marker genes in each cell group, and size indicates the proportion of cells expressing marker genes. (C) Z-score normalized expression of *Cdc42* in WT thymocyte subsets (DN1–DN3, DP1–DP3, 4SP, and 8SP) based on single-cell RNA-seq data.

Figure S8. Regulon analysis of total thymocytes

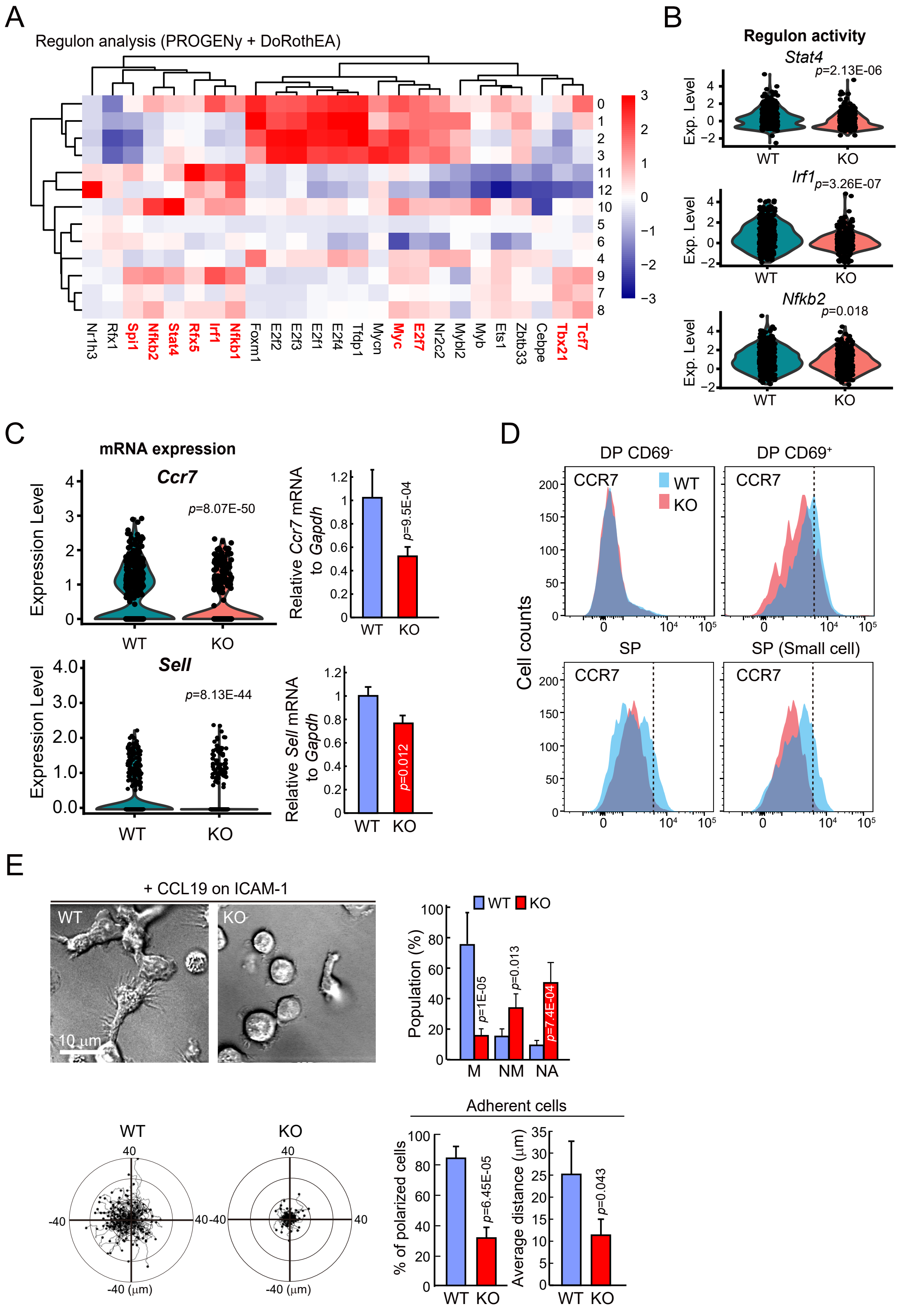

Figure S8. Regulon analysis of total thymocytes. (A) Hierarchical clustering of Regulon analysis from 13 clusters. DoRothEA database and PROGENy method were combined for analysis. The red font indicates the regulons whose expression has increased in Cluster 8 (SP stage). (B) Violin plots of regulon activity of *Stat4*, *Irf1* and *Nfkb2* from Cluster 8 (SP stage). (C) Violin plots of *Ccr7* and *Sell* mRNA expression from Cluster 8 (SP stage) depicted in Fig. 3A and the real-time quantitative PCR analysis for validation. (D) Flow cytometric analysis of CCR7 expression in $CD4^+$ single-positive (SP) thymocytes. Histograms compare the expression levels of CCR7 between *Cdc42*$^{f/f}$ and *Cdc42*$^{f/f}$CD4Cre cells in both DP $CD69^+$ and SP (small cell) populations, highlighting differences in surface marker expression. (E) Chemokinesis assay. $CD4^+$ SP thymocytes were placed on ICAM-1 coated plate, stimulated with CCL19, and imaged for 20 min under EVOS microscopy. M, motile cells; NM, non-motile cells; NA, non-adherent cells. Cell trace analysis of $CD4^+$ SP thymocytes were analyzed by Imaris 8.0 software. Statistics was performed using student's t test.

Figure S9. Comprehensive gene profiling involved in vesicle generation

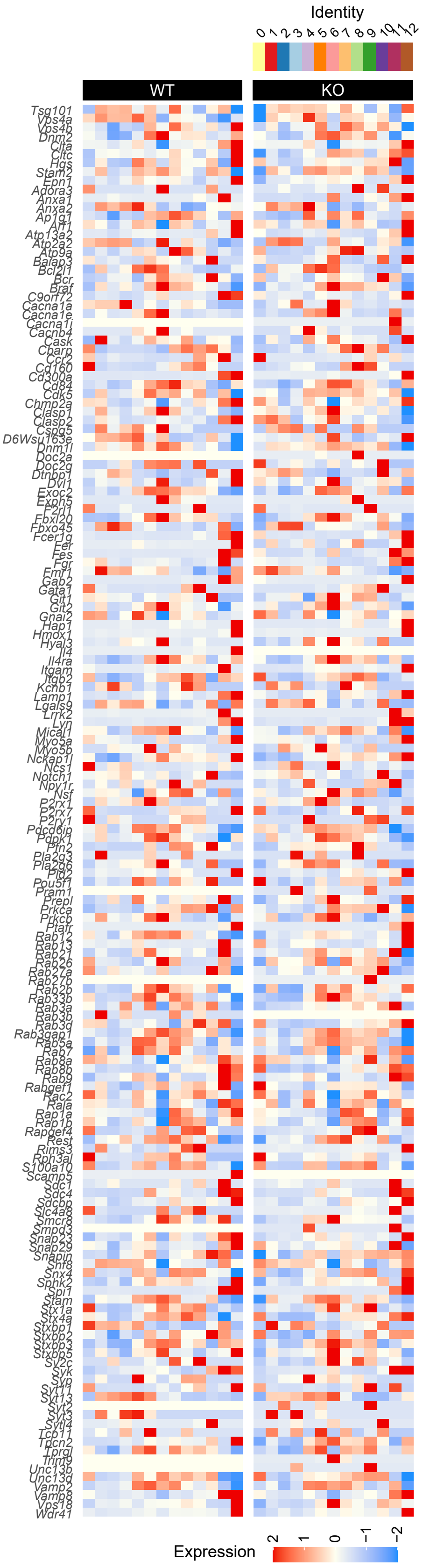

Figure S9. Comprehensive gene profiling involved in vesicle generation. (A) Heatmap showing the expression levels of genes related to vesicle generation in WT and *Cdc42*$^{f/f}$CD4Cre-KO thymocytes. Single-cell RNA sequencing (scRNA-seq) data were analyzed and grouped into 13 clusters (color bar at the top) representing different thymocyte subsets. Each row corresponds to a gene involved in vesicle formation, trafficking, and exocytosis, while each column represents an individual cell.

Figure S10. CD4⁺ T cells inhibiting Cdc42 exhibit a severe defect in adhesion to high endothelial venules in vivo

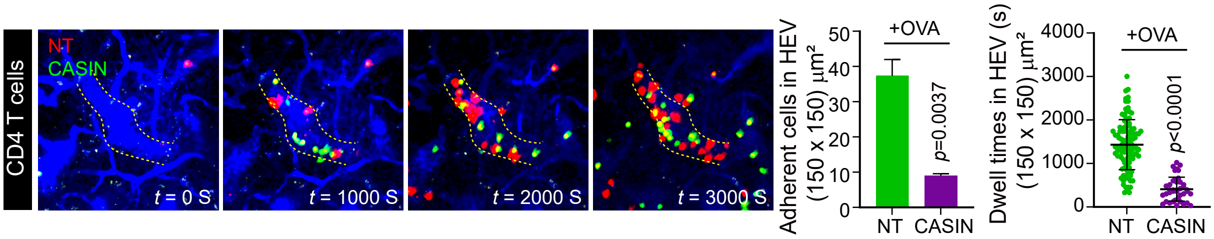

Figure S10. $CD4^+$ T cells inhibiting Cdc42 exhibit a severe defect in adhesion to high endothelial venules *in vivo*. In vivo two-photon imaging of HEV interactions by OT-II $CD4^+$ T cells (with or without CASIN) for 3000 s. Representative time-lapse images show the dynamics of cell adhesion and accumulation within HEVs (outlined in yellow). Quantification of adherent cells per 150 × 150 μm^2 field (left) and dwell times within HEVs (right) are shown. Data represent the mean ± SEM of three independent experiments. Statistical significance was determined using unpaired Student's *t* test.

Movie S1 (separate file). TCR clustering of $CD4^+$ SP thymocytes in the supported lipid bilayer. $CD4^+$ SP thymocytes obtained from the thymus of OTII crossed *Cdc42*$^{f/f}$ and *Cdc42*$^{f/f}$CD4Cre mice were stained with anti-TCRβ (H57Fab-Alexa594) and stimulated on supported lipid bilayer with $OVA_{323-339}$/I-A^b and ICAM-1. Centripetal movement of TCR microclusters was imaged for 20 min every 12s using a TIRF microscope. This video corresponds to Figure 4B.

Movie S2 (separate file). $CD4^+$ SP thymocytes from *Cdc42* KO mice exhibit severe defect in adhesion to HEVs *in vivo*. Two-photon intravital imaging of $CD4^+$ SP thymocytes at the HEVs of the popliteal lymph node. $CD4^+$ SP thymocytes from *Cdc42*$^{f/f}$ OT-II (WT) and *Cdc42*$^{f/f}$ CD4-Cre OT-II (KO) mice were labeled with CMRA (orange) and CMFDA (green), respectively, and intravenously injected into WT recipient mice. The recipients had been injected 24 h earlier with $OVA_{323-339}$-pulsed BMDCs. HEVs were visualized by intravenous injection of Dextran-Cascade Blue™. The popliteal lymph node was imaged using two-photon microscopy for 3 h.
This video corresponds to Figure 7A.

Movie S3 (separate file). *Cdc42*-KO $CD4^+$ SP thymocytes move faster than wild-type $CD4^+$ SP thymocytes at the popliteal lymph node (Site I). Two-photon microscope imaging of $CD4^+$ SP thymocytes at popliteal lymph node. CMRA-labelled and $OVA_{323-339}$-pulsed BMDCs were injected to footpad of recipient wild-type mice. CMFDA-labelled $CD4^+$ SP thymocytes (OTII crossed *Cdc42*$^{f/f}$CD4Cre) were *i.v.* injected at 24 h post DC injection and popliteal lymph node was imaged for 2 h at 24 h post injection of $CD4^+$ SP thymocytes. Two migrating cells (non-interacting cells) were randomly selected in each condition, highlighted as white and red dot circle, and tracked for 35 min. A track path was visualized as dot line. This video corresponds to Figure 7C.

Movie S4 (separate file). Reduced interaction of $CD4^+$ SP thymocytes from *Cdc42* KO mouse with antigen-pulsed DCs at the popliteal lymph node (Site II). Two-photon microscope imaging of $CD4^+$ SP thymocytes interacting with antigen-pulsed DCs at popliteal lymph node. CMRA-labelled and $OVA_{323-339}$-pulsed BMDCs were injected to footpad of recipient wild-type mice. CMFDA-labelled $CD4^+$ SP thymocytes (OTII crossed Cdc42$^{f/f}$CD4Cre) were *i.v.* injected at 24 h post DC injection and popliteal lymph node was imaged for 2 h at 24 h post injection of $CD4^+$ SP thymocytes. Two DC-interacting T cells were randomly selected in each condition, highlighted as white and red dot circle, and tracked for 35 min. A track path was visualized as dot line. This video corresponds to Figure 7D.

Movie S5 (separate file). CASIN-treated OTII $CD4^+$ T cells exhibit a severe defect in adhesion to HEVs in vivo. OTII $CD4^+$ T cells, pretreated with or without CASIN, were labeled with CMRA (orange) or CMFDA (green), respectively, and intravenously injected into wild-type recipient mice that had received $OVA_{323-339}$-pulsed BMDCs 24 h earlier. HEVs were visualized using Dextran–Cascade Blue™. The popliteal lymph node was imaged by two-photon microscopy up to 3000s. This video corresponds to Figure S10.

SI References

Experiment References:

1. H. Kim, *et al.*, T cell microvilli constitute immunological synaptosomes that carry messages to antigen-presenting cells. *Nat. Commun.* **9**, 3630–3648 (2018).
